# miR-223 alleviates DSS-induced colitis by prompting macrophage M2 polarization through PPAR-γ/FOXO1 signaling

**DOI:** 10.3389/fimmu.2025.1598781

**Published:** 2025-07-29

**Authors:** Juanjuan Zhang, Lixin Wang, Yingye Shen, Xiaoli Qian, Zhiming Wang, Chenyang Wang

**Affiliations:** Key Laboratory of Intestinal Damage, Research Institute of General Surgery, Jinling Hospital, Affiliated Hospital of Medical School, Nanjing University, Nanjing, Jiangsu, China

**Keywords:** miR-223, DSS-induced colitis, intestinal macrophages, macrophage polarization, inflammatory bowel disease (IBD)

## Abstract

**Background:**

Macrophage polarization represents a promising therapeutic target for inflammatory bowel disease (IBD). This study investigates the role of miRNA-223 (miR-223) in dextran sodium sulfate (DSS)-induced colitis and its regulation of macrophage polarization.

**Methods:**

Male C57BL/6 mice were assigned to four groups: Wild-type (WT) control, DSS-treated group (DSS), DSS+miR-223 agomir (DSS+A), and DSS+ miR-223 agomir negative control (DSS+NC). Colitis was induced with 2.5% DSS for 7 days; miR-223 agomir or NC was administered intraperitoneally on days 2–4. We evaluated disease activity index (DAI), colonic inflammation, and the expression of inflammatory mediators, peroxisome proliferator-activated receptor gamma (PPAR-γ) and forkhead box transcription factor O1 (FOXO1).

**Results:**

Histopathological analysis showed that miR-223 agomir significantly attenuated DSS-induced colon damage. Proinflammatory cytokines (TNF-α, IL-1β, IL-6) increased in DSS mice, while anti-inflammatory IL-10 decreased—trends reversed by miR-223 supplementation at mRNA/protein levels. Mechanistically, DSS elevated M1 macrophage marker iNOS and FOXO1 but reduced M2 marker Arg-1 and PPAR-γ. miR-223 agomir suppressed M1 polarization while enhancing M2 polarization by downregulating FOXO1 and upregulating PPAR-γ.

**Conclusion:**

We identify a novel dual-regulatory mechanism wherein miR-223 ameliorates colitis by shifting macrophage polarization from M1 to M2 *via* concurrent FOXO1 suppression and PPAR-γ activation. These findings establish a mechanistic basis for miR-223 supplementation as a novel IBD therapeutic strategy.

## Introduction

Inflammatory bowel disease (IBD), encompassing Crohn’s disease (CD) and ulcerative colitis (UC), is characterized by chronic immune dysregulation involving complex interactions between genetic susceptibility and the mucosal microenvironment (1). Intestinal macrophages—sentinel immune cells central to IBD pathogenesis—paradoxically sustain both tissue homeostasis and inflammatory cascades through dynamic polarization states (2). These cells exist along a continuum from pro-inflammatory M1 phenotypes (marked by iNOS and CD86) to anti-inflammatory M2 subtypes (expressing Arg1 and CD206), with their balance dictating disease progression (3). Recent therapeutic paradigms highlight macrophage polarization as a pivotal control point: M1 dominance correlates with epithelial barrier disruption *via* TNF-α/IL-1β overproduction, while M2 skewing promotes mucosal healing through IL-10/TGF-β secretion (4–6). Consequently, epigenetic regulators of phenotypic plasticity, particularly microRNA-mediated macrophage reprogramming, represent an emerging frontier in colitis management.

As master regulators of post-transcriptional gene networks, microRNAs (miRNAs) orchestrate immune homeostasis by epigenetic fine-tuning of myeloid cell differentiation and inflammatory responses (7). Among these regulators, the myeloid-specific miR-223 functions as an immunological rheostat—its dysregulation directly correlates with pathological inflammation through modulation of neutrophil chemotaxis, inflammasome activation, and crucially, macrophage polarization plasticity (8, 9). While current evidence indicates miR-223 promotes anti-inflammatory M2 macrophage polarization in sepsis and metabolic disorders (10, 11), its transcriptional regulation of intestinal macrophage polarization remains poorly defined. This knowledge gap is particularly significant regarding miR-223’s coordination of dual targets in IBD pathogenesis. Such mechanistic insight is pivotal given that optimal macrophage reprogramming requires simultaneous suppression of pro-inflammatory pathways (*e.g.*, FOXO1) and activation of resolution signals (*e.g.*, PPAR-γ)—a combinatorial regulatory paradigm unexplored in miRNA-mediated colitis therapy.

While accumulating evidence demonstrates miR-223’s anti-inflammatory properties in colitis models (12–15), its mechanistic regulation of intestinal macrophage polarization remains a critical gap in IBD research. Building on our prior findings, we propose that miR-223 ameliorates colitis by coordinately regulating PPAR-γ and FOXO1 to reprogram macrophage polarization. In the present study, we systematically investigate how miR-223 coordinates the PPAR-γ/FOXO1 signaling axis to reprogram macrophage phenotypes in DSS-induced colitis—an unexplored therapeutic nexus bridging epigenetic regulation and mucosal immunity. Using multimodal approaches (histopathology, RT-qPCR, WB, ELISA & immunofluorescence), we establish miR-223’s dual regulatory role: suppressing pro-inflammatory M1 polarization through FOXO1 inhibition while amplifying M2 resolution *via* PPAR-γ activation. This work provides the first mechanistic blueprint for miRNA-directed macrophage plasticity in intestinal inflammation.

## Materials and methods

### Mice and groups

**Figure 1:**
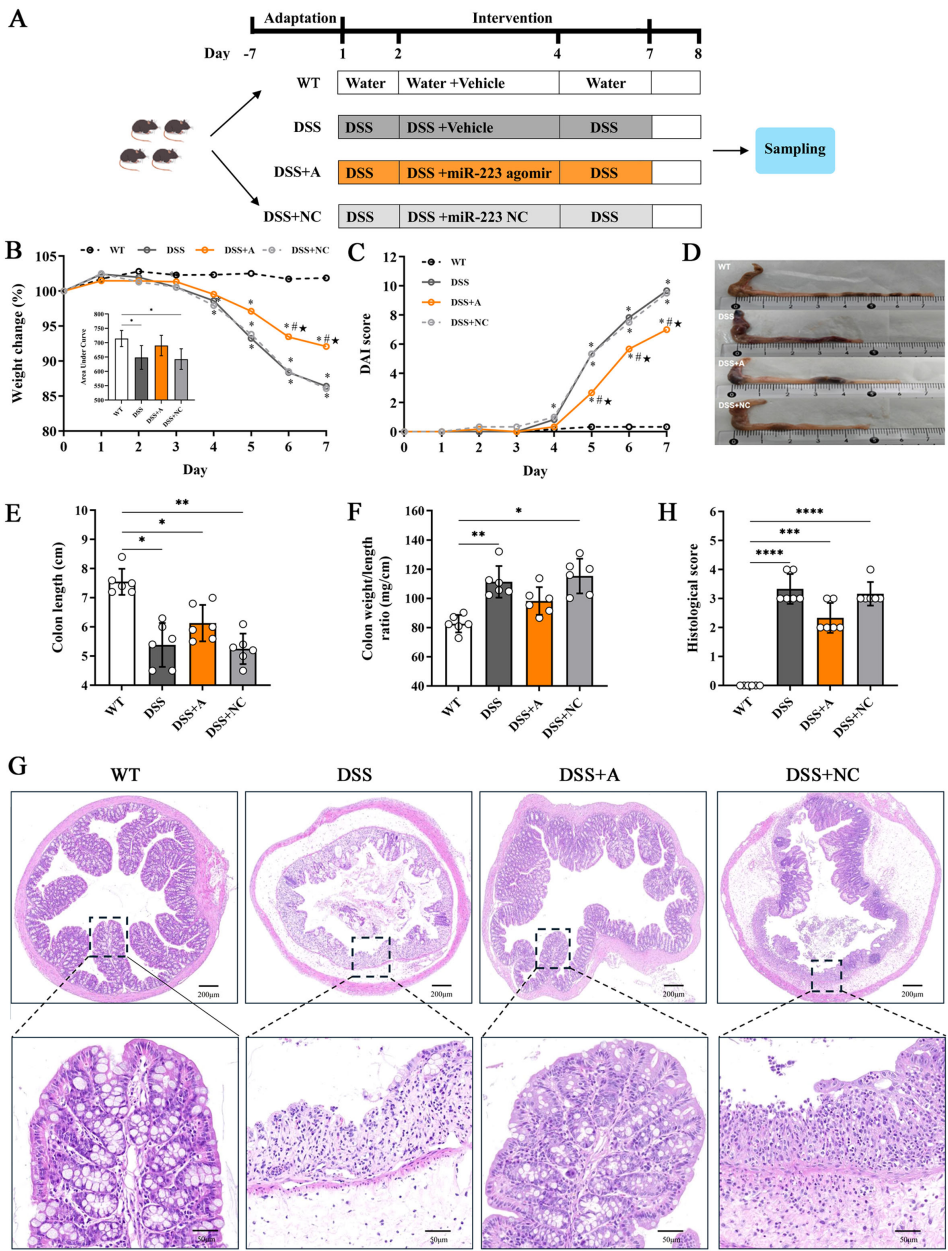
miR-223 alleviated intestinal inflammation in a DSS-induced murine model of colitis. **(A)** Description of the experimental design. C57BL/6J mice were randomly divided into four groups: Wild-type (WT) control, DSS-treated group (DSS), DSS+miR-223 agomir (DSS+A), and DSS+ miR-223 agomir negative control (DSS+NC) (n=6 each group). The mice in the DSS, DSS+A and DSS+NC groups were exposed to 2.5% dextran sodium sulfate (DSS) for 7 days. The experimental groups were intraperitoneally administered sterile PBS, the miR-223 agomir or the miR-223 agomir negative control on days 2, 3 and 4, respectively. WT: wild type. **(B)** Dynamic alterations in the daily body weight (%) of the mice. The body weights on day 0 were recorded as the baseline (100%) (n=6 each group). Inset: Area under curve (AUC) analysis of cumulative weight loss. **(C)** Changes in the disease activity index (DAI) (n=6 each group). **(D)** Representative macroscopic images of colons. **(E)** Comparisons of colon length among the groups. All the data are expressed as the means ± SDs (n=6 each group). *p<0.05, **p<0.01. **(F)** Changes in the ratios (mg/cm) between the wet weight of the colon and the length of the colon. All the data are expressed as the means ± SDs (n=6 each group). *p<0.05, **p<0.01. **(G)** Representative micrographs showing colon tissues stained with H&E. Images were taken at magnifications of 4× (above panel) and 20× (below panel). **(H)** Histological score. All the data are expressed as the means ± SDs (n=6 each group). *p<0.05, **p<0.01, ***p<0.001, ****p<0.0001.

Twenty-four male C57BL/6 mice (6~8 weeks old) were obtained from GemPharmatech Co., Ltd. (Nanjing, China). All mice were housed under specific pathogen-free (SPF) conditions with controlled temperature, humidity, and ventilation. All procedures complied with Chinese animal welfare regulations and were approved by the Ethics Committee of the Jinling Hospital, Affiliated Hospital of Medical School, Nanjing University (Approval No. 2022DZGKJDWLS-0072). Mice were randomly assigned to four groups (n=6/group): Wild-type (WT) control, DSS-treated group (DSS), DSS+miR-223 agomir (DSS+A), and DSS+ miR-223 agomir negative control (DSS+NC) (**Figure 1A**).

### DSS-induced colitis model

All groups except WT received 2.5% dextran sulfate sodium (DSS; MP Biomedicals, Shanghai, China) dissolved in drinking water *ad libitum* for 7 days to induce colitis. The DSS solution was refreshed every 48 hours. Daily monitoring included body weight, stool consistency and fecal blood, with colitis disease activity index (DAI) scores calculated according to established criteria (16). The DAI quantifies colitis severity through three clinical parameters: weight loss percentage, stool consistency score, and fecal blood presence.

### miR-223 agomir administration

To overexpress miR-223 *in vivo*, mice received daily intraperitoneal (*i.p.*) injections of a synthetic, chemically modified double-stranded miR-223 agomir (Genepharma, Shanghai, China) or its negative control agomir (NC agomir), both designed as 22-nt RNA oligos. The miR-223 agomir sequence was 5’-UGUCAGUUUGUCAAAUA CCCCA-3’, and the NC agomir sequence was 5’-UUCUCCGAACGUGUCACGUTT-3’. The agomirs were reconstituted in sterile RNase-free phosphate-buffered saline (PBS) to a stock concentration of 3 mg/mL. They were then administered intraperitoneally at a dose of 1.5 mg/kg body weight per day as described previously (12). Beginning 24 hours after DSS induction, mice received daily *i.p.* injections for three consecutive days, with each dose diluted into a final volume of 100 µL sterile RNase-free PBS immediately prior to injection.

### Sampling

On day 8, all mice were humanely euthanized by CO_2_ asphyxiation following isoflurane anesthesia (4% induction, 1.5–2% maintenance in oxygen). This protocol adhered to international ethical standards for minimizing distress. Colons were immediately excised and measured for weight and length to assess inflammatory status. Proximal colon segments were fixed for histopathological analysis. Remaining tissues were snap-frozen in liquid nitrogen and stored at -80°C for subsequent molecular analyses, including immunofluorescence, western blotting (WB), and quantitative real-time PCR (RT-qPCR).

### Histological assessment of colitis

Colonic tissues were excised, washed with ice-cold PBS, fixed in 10% neutral buffered formalin, and embedded in paraffin. Sequential 5-µm sections were prepared and stained with hematoxylin and eosin (H&E) for histological evaluation. Two independent pathologists, blinded to experimental conditions, scored tissue samples according to established histological criteria (17). For specialized assessment of mucin-producing cells, proximal colon samples were fixed in Carnoy’s solution and stained with alcian blue (AB). Five randomly selected microscopic fields per AB-stained section were quantitatively analyzed to evaluate goblet cell distribution.

### RNA extraction and qPCR

Total RNA from colonic tissues was extracted *via* a TaKaRa MiniBEST Universal RNA Extraction Kit (Cat.# 9767, TaKaRa Bio Inc., Dalian, Liaoning, China) according to the manufacturer’s instructions. The RNA concentration was quantified *via* a NanoDrop 2000 (Thermo Fisher Scientific, Inc., Waltham, MA, USA). Reverse transcription PCR (RT–PCR) was performed to convert RNA into complementary DNA (cDNA) *via* PrimeScript™ RT Master Mix (Cat.# RR036A, TaKaRa Bio Inc.) on a GeneAmp^®^ PCR system 9700 (S/N 805S3242413,Applied Biosystem, Foster City, CA, USA). qPCR was conducted on an ABI 7500 system (S/N 275008197, Applied Biosystems) with TB Green^®^ Premix Ex Taq II (Tli RNaseH Plus) (Cat.# RR820A, TaKaRa Bio Inc.) with the appropriate primer sets (**Table 1**). For miR-223 quantification, cDNA synthesis was performed using Bulge-Loop™ miRNA-specific stem-loop primers (Cat.# Q1124, RiboBio, Guangzhou, China). Primer sets for miR-223-3p (including one reverse transcription primer and paired qPCR primers) were custom-designed by RiboBio. Total RNA (1 µg) was reverse-transcribed into cDNA with the FastKing RT kit (Cat.# A0830A, TIANGEN Biotech, Beijing, China), followed by qPCR under standard cycling conditions. All reactions were performed in technical triplicate. Expression levels of target genes were normalized to *Gapdh* mRNA, while miR-223 was normalized to U6 snRNA. Both were quantified using the 2^−ΔΔCt^ method.

**Table 1:** Primer sequence for quantitative PCR.

Gene	Primer Sequence (5' to 3')	
*Tnf-α*	Forward	TCTTCTCATTCCTGCTTGTGG
	Reverse	GGTCTGGGCCATAGAACTGA
*Il-6*	Forward	GCTACCAAACTGGATATAATCAGGA
	Reverse	CCAGGTAGCTATGGTACTCCAGAA
*Il-1β*	Forward	GAAATGCCACCTTTTGACAGTG
	Reverse	TGGATGCTCTCATCAGGACAG
*Il-10*	Forward	GGTCTGAGTGGGACTCAAGG
	Reverse	CGTGGCAATGATCTCAACAC
*iNos*	Forward	CAAGCACCTTGGAAGAGGAG
	Reverse	AAGGCCAAACACAGCATACC
*Arg-1*	Forward	CAGAAGAATGGAAGAGTCAG
	Reverse	CAGATATGCAGGGAGTCACC
*Foxo1*	Forward	AGATGAGTGCCCTGGGCAGC
	Reverse	GATGGACTCCATGTCACAGT
*Ppar-γ*	Forward	GGAAAGACAACGGACAAATCAC
	Reverse	TACGGATCGAAACTGGCAC
*Gapdh*	Forward	GGTTGTCTCCTGCGACTTCA
	Reverse	TGGTCCAGGGTTTCTTACTCC

### Western blotting analysis

Colonic tissue samples (50 mg) were homogenized in 0.5 mL of ice-cold RIPA buffer (Cat.# R0278, Sigma–Aldrich, St. Louis, USA) supplemented with a complete protease inhibitor cocktail (Cat.# P8340, Sigma–Aldrich). Following homogenization, the lysate was maintained on ice for 45 min to facilitate complete lysis. Subsequently, the mixture was centrifuged at 4°C for 5 min (13,000 × *g*) to pellet cellular debris and collect the protein-rich supernatant. The resulting protein-containing supernatant was aliquoted and cryopreserved at −80°C for further analysis. Protein concentrations were quantified using a bicinchoninic acid (BCA) assay kit (Cat.# 23225, Thermo Fisher, USA). Proteins were resolved by electrophoresis on 12% SDS-polyacrylamide gels and subsequently transferred to PVDF membranes (0.45 µm; Amersham Biosciences, USA). Post-transfer, membranes were blocked with 10% nonfat dry milk at room temperature and then incubated overnight at 4°C with anti-Arg-1 antibody (1:3000) (Cat.# 16001-1-AP, Proteintech, Wuhan, China), anti-iNOS antibody (1:1000) (Cat.# 18985-1-AP, Proteintech), anti-PPAR-γ antibody (1:3000) (Cat.# 16643-1-AP, Proteintech), anti-FOXO1 antibody (1:1000) (Cat.# 18592-1-AP, Proteintech) and anti-β-actin antibody (1:5000) (Cat.# 66009-1-Ig, Proteintech). The HRP-conjugated anti-rabbit (Cat.# A21020, Abbkine, Atlanta, USA) or mouse (Cat.# A21010, Abbkine) secondary antibody was added after washing with Tris-buffered saline with Tween 20 (TBST). The expression levels of the proteins are presented as percentages (%) relative to β-actin expression, and the intensities of the bands were calculated with ImageJ software (National Institutes of Health, USA).

### Immunofluorescence staining

For the paraffin sections, dewaxing, hydration, and antigen retrieval steps were performed according to standard protocols. After being blocked with bovine serum albumin blocking buffer (3% in PBS), the slides were incubated with the following primary antibodies: anti-PPAR-γ antibody (1:3000) (Cat.# GB11163, Servicebio, Wuhai, China), anti-FOXO1 antibody (1:10000) (Cat.# GB11286-1, Servicebio), anti-CD206 (1:5000) (Cat.# GB113497, Servicebio), anti-CD86 (1:5000) (Cat.# GB115630, Servicebio) or anti-F4/80 (1:2000) (Cat.# GB113373, Servicebio) overnight at 4°C. The slides were then incubated with a secondary antibody (HRP-conjugated goat anti-rabbit or -mouse IgG) for 1 h at room temperature. Antibodies were diluted in PBS (pH 7.4). A solution (1:500) containing If555-tyramide (Cat.# G1233, Servicebio) or iF488-tyramide (Cat.# G1231, Servicebio) was used for immunofluorescence staining. DAPI (Cat.# 62248, Thermo Fisher Scientific, 1:10000 dilution) was added to counterstain the cell nuclei on the glass slides. Confocal imaging was performed *via* a TCS SP5 II system (S/N 5100001093, Leica, Wetzlar, Germany), and images were processed *via* the Leica AF software suite. Five different views in each section were captured. The integral optical density (IOD) and positive area/cells were calculated *via* ImageJ Pro Plus.

### Enzyme-linked immunosorbent assay

The protein supernatants from the colonic tissues were diluted in ice-cold PBS containing a protease inhibitor cocktail and then used for assessment of cytokine levels. The levels of myeloperoxidase (MPO) (Cat.# EK2133, LiankeBio, Hangzhou, China), TNF-α (Cat.# EK282HS, LiankeBio), IL-1β (Cat.# EK201BHS, LiankeBio), IL-6 (Cat.# EK206, LiankeBio) and IL-10 (Cat.# EK210, LiankeBio) in the supernatant were quantified *via* ELISA kits according to the manufacturer’s protocols.

### Statistical analyses

Statistical analyses were performed using GraphPad Prism (version 9.3.1). Experimental data are presented as means ± standard deviations (SD). Differences between groups were assessed using one-way analysis of variance (ANOVA) followed by Tukey’s multiple comparison test for *post hoc* analysis. Area under the curve (AUC) analysis was used to compare longitudinal changes in body weight across groups. Pearson correlation analysis was employed to evaluate the relationships between miR-223 expression and levels of colonic inflammatory mediators or macrophage phenotypes. Statistical significance was defined as p < 0.05.

## Results

### DSS successfully induced acute ulcerative colitis with characteristic pathophysiological alterations

**Figure 2:**
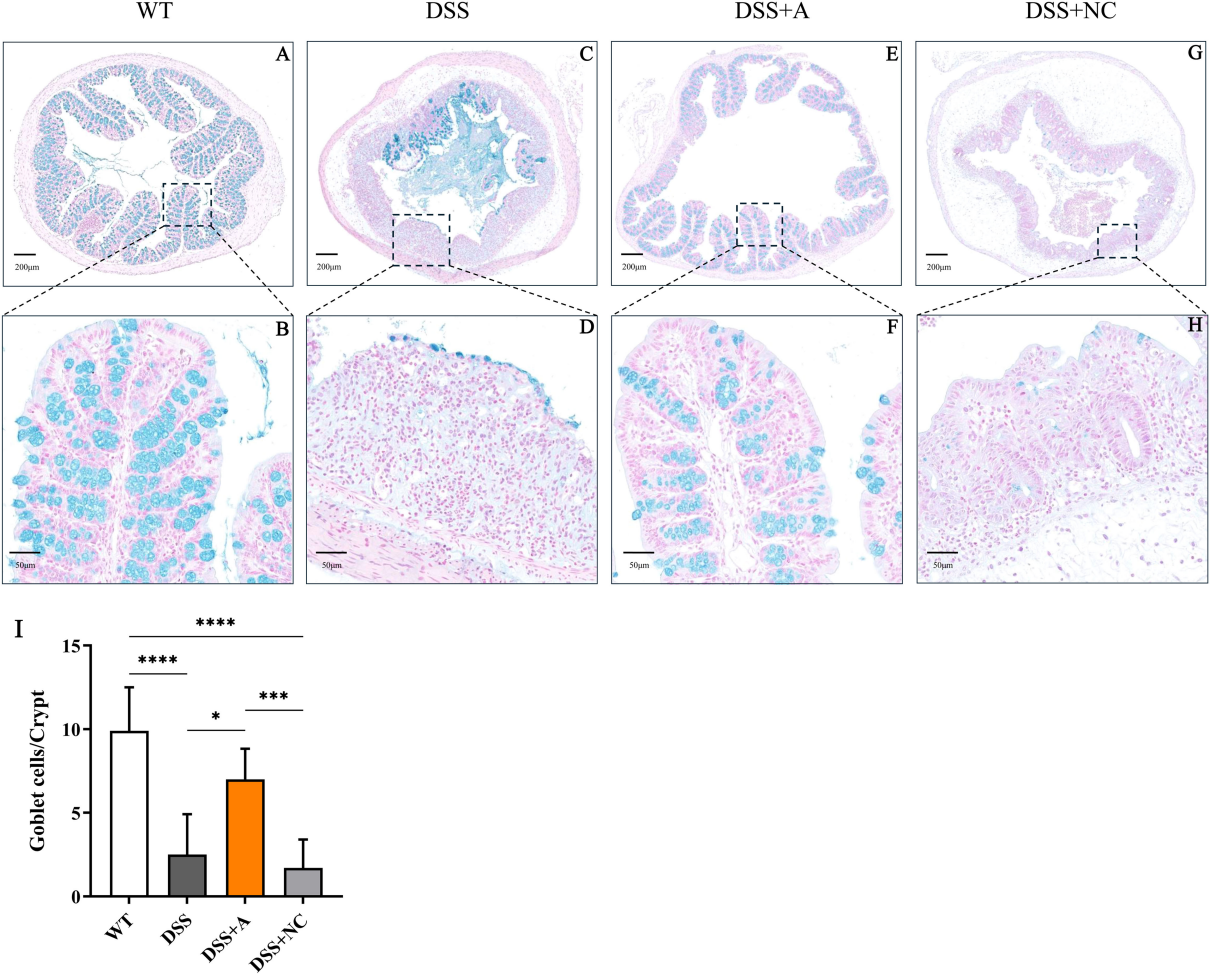
Representative images showing colonic tissues stained with Alcian blue. Images were taken at magnifications of 4× **(A, C, E, G)** and 20× **(B, D, F, H)**. **(I)** Numbers of goblet cells in each crypt. All the data are expressed as the means ± SDs (n=10 HPFs per group). *p<0.05, ***p<0.001, ****p<0.0001.

Administration of 2.5% DSS for 7 days elicited robust colitis progression, as evidenced by progressive weight loss (13.6% mean reduction *vs* WT, p<0.01), elevated disease activity index (DAI: 9.67 ± 0.4 *vs* 0.33 ± 0.1 in WT, *p*<0.001), and bloody diarrhea in DSS-exposed groups (**Figures 1B, C**). Pathological features peaked at day 7, characterized by colon shortening (5.38 ± 0.75 cm *vs* 7.55 ± 0.45 cm in WT, p<0.05) and increased colon weight/length ratio—an indicator of edema and inflammation (111.4 ± 10.8 mg/cm *vs* 82.7 ± 5.9 mg/cm, p<0.01) in DSS-treated mice (**Figures 1D-F**). Histopathological analysis of colonic tissues by H&E staining showed a marked elevation in the inflammatory score in the DSS group (3.33 ± 0.52 *vs* 0.00 ± 0.00 in WT, p<0.01) (**Figures 1G, H**). Additionally, the DSS group exhibited severe epithelial mucosal loss, glandular atrophy, and significant reduction in the number of goblet cells within the colon (**Figures 2A, B**).

### miR-223 supplementation attenuated DSS−induced colitis

Administration of the miR-223 agomir (DSS+A group) significantly mitigated clinical and pathological features of colitis in mice. Mice treated with miR-223 agomir exhibited less weight loss than the DSS group, achieving significantly higher body weights starting on day 5 (**Figure 1B**). Supporting this finding, AUC analysis of the longitudinal weight data showed no significant difference between the DSS+A group and the WT group (**Figure 1B**, inset). Furthermore, the onset of bloody stools—a hallmark of colitis—was delayed in the DSS+A group, contributing to a significant decrease in DAI scores from day 5 onward (**Figure 1C**). Notably, these improvements in weight stability, clinical symptoms, and DAI scores were also statistically significant when comparing the DSS+A group to the negative control (DSS+NC) group, underscoring the specificity of miR-223’s therapeutic effect. Postmortem analyses revealed pronounced morphological improvements in the DSS+A group. Colon lengths were significantly preserved compared to both the DSS and DSS+NC groups (**Figure 1D**), while the colon weight/length ratio was markedly reduced (**Figures 1E, F**). Histological evaluation of H&E-stained colon tissues demonstrated diminished inflammatory cell infiltration and tissue damage in the DSS+A group, as evidenced by lower histological inflammatory scores relative to the DSS group (**Figure 1G**). In addition, Alcian blue staining showed a higher density of goblet cells in the DSS+A group compared to the DSS group (**Figure 2**), suggesting enhanced mucosal protection and epithelial repair following miR-223 supplementation. Collectively, these findings indicated that miR-223 agomir administration could alleviate DSS-induced colitis by ameliorating clinical symptoms, preserving colon morphology, and promoting mucosal healing.

### miR-223 supplementation alleviated DSS-induced colonic inflammation

**Figure 3:**
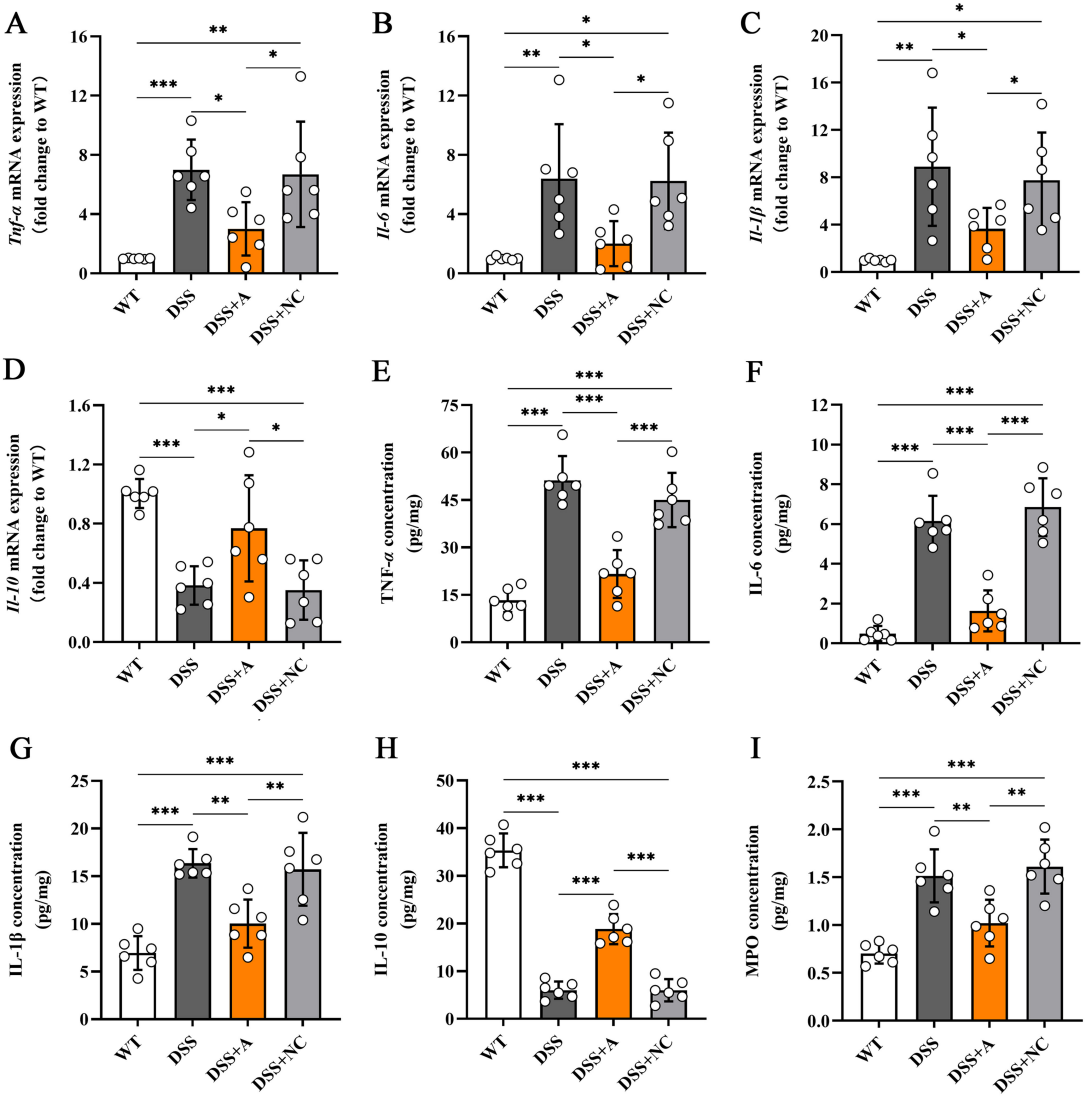
Effects of miR-223 supplementation on inflammatory mediators in colonic tissues. The expression levels of inflammatory mediators were measured *via* RT–qPCR and ELISA (n=6 each group). **(A-D)** Variations in the mRNA expression of colonic TNF-α, IL-6, IL-1β, and IL-10. **(E-I)** Changes in the colonic contents of TNF-α, IL-6, IL-1β, IL-10 and MPO. All the data are expressed as the means ± SDs (n=6 each group). *p<0.05, **p<0.01, ***p<0.001, ****p<0.0001. miR, microRNA; mRNA, messenger RNA; TNF-α, tumor necrosis factor-α; IL, interleukin; MPO, myeloperoxidase; RT–qPCR, quantitative reverse transcription PCR; ELISA, enzyme-linked immunosorbent assay.

To evaluate the anti-inflammatory effects of miR-223, we quantified key inflammatory mediators in colon tissues *via* RT-qPCR and ELISA. Compared to the wild-type (WT) group, mice in the DSS group exhibited a pronounced inflammatory response, characterized by elevated levels of pro-inflammatory cytokines (TNF-α, IL-1β, IL-6) (**Figures 3A-C, E-G**) and myeloperoxidase (MPO)—a marker of neutrophil infiltration (**Figure 3I**), alongside reduced levels of the anti-inflammatory cytokine IL-10 (**Figures 3D, H**). Notably, miR-223 agomir administration (DSS+A group) reversed these inflammatory perturbations. Relative to the DSS group, the DSS+A group showed significantly lower colonic MPO concentration and reduced TNF-α, IL-1β, and IL-6 levels, indicating suppressed neutrophil recruitment and pro-inflammatory signaling (**Figures 3A-C, E-G**). Concurrently, IL-10 expression in colon tissues was restored to levels approaching those of the WT group, suggesting a reinstated anti-inflammatory counterbalance (**Figures 3D, H**). The data demonstrated that miR-223 supplementation could mitigate DSS-driven colonic inflammation by dampening pro-inflammatory pathways while enhancing IL-10-mediated resolution, further supporting its therapeutic potential in colitis.

### miR-223 exerted anti-inflammatory effects in DSS-induced colitis *via* cytokine modulation

**Figure 4:**
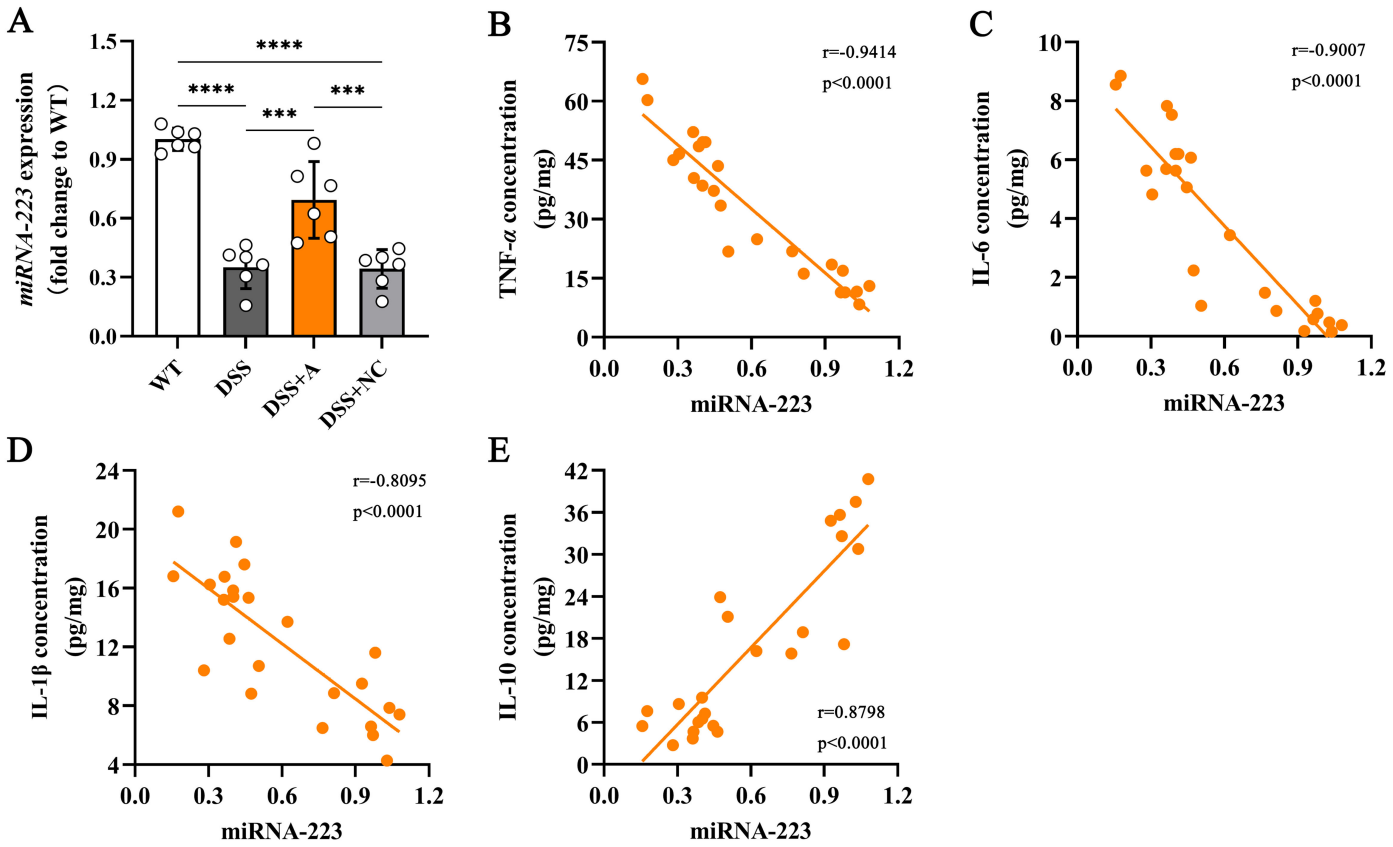
Correlations between colonic miR-223 and inflammatory mediators. **(A)** The expression of colonic miR-223 in different groups. All the data are expressed as the means ± SDs (n=6 each group). ***p<0.001, ****p<0.0001. **(B-E)** Association of colonic miR-223 expression with TNF-α, IL-6, IL-1β and IL-10 in the colon. Pearson correlation analyses were performed to investigate the relationships between miR-223 and colonic inflammatory mediators. TNF-α, tumor necrosis factor-α; IL, interleukin.

To elucidate the mechanistic role of miR-223 in mitigating colonic inflammation, we analyzed its expression dynamics and relationships with inflammatory mediators. Colonic miR-223 levels were significantly reduced in the DSS group compared to the wild-type (WT) group, confirming its dysregulation during colitis (**Figure 4A**). Conversely, miR-223 agomir treatment (DSS+A group) restored colonic miR-223 expression to levels exceeding those in the DSS group (**Figure 4A**), demonstrating successful therapeutic targeting. Strikingly, correlation analyses revealed robust associations between miR-223 and inflammatory cytokines. miR-223 expression exhibited strong negative correlations with pro-inflammatory mediators, including TNF-α (r = -0.9414, p < 0.0001), IL-1β (r = -0.8095, p < 0.0001), and IL-6 (r = -0.9007, p < 0.0001) (**Figures 4B–D**). In contrast, miR-223 expression showed a positive correlation with the anti-inflammatory cytokine IL-10 (r = 0.8798, p < 0.0001) (**Figure 4E**). The results suggested that miR-223 could alleviate colitis by coordinately suppressing pro-inflammatory cytokine production and enhancing IL-10-mediated anti-inflammatory signaling. Together, these data positioned miR-223 as a critical regulator of immune balance in colitis, with its downregulation exacerbating inflammation and its restoration counteracting disease-associated cytokine dysregulation.

### miR-223 modulated macrophage polarization in DSS-induced colitis

**Figure 5:**
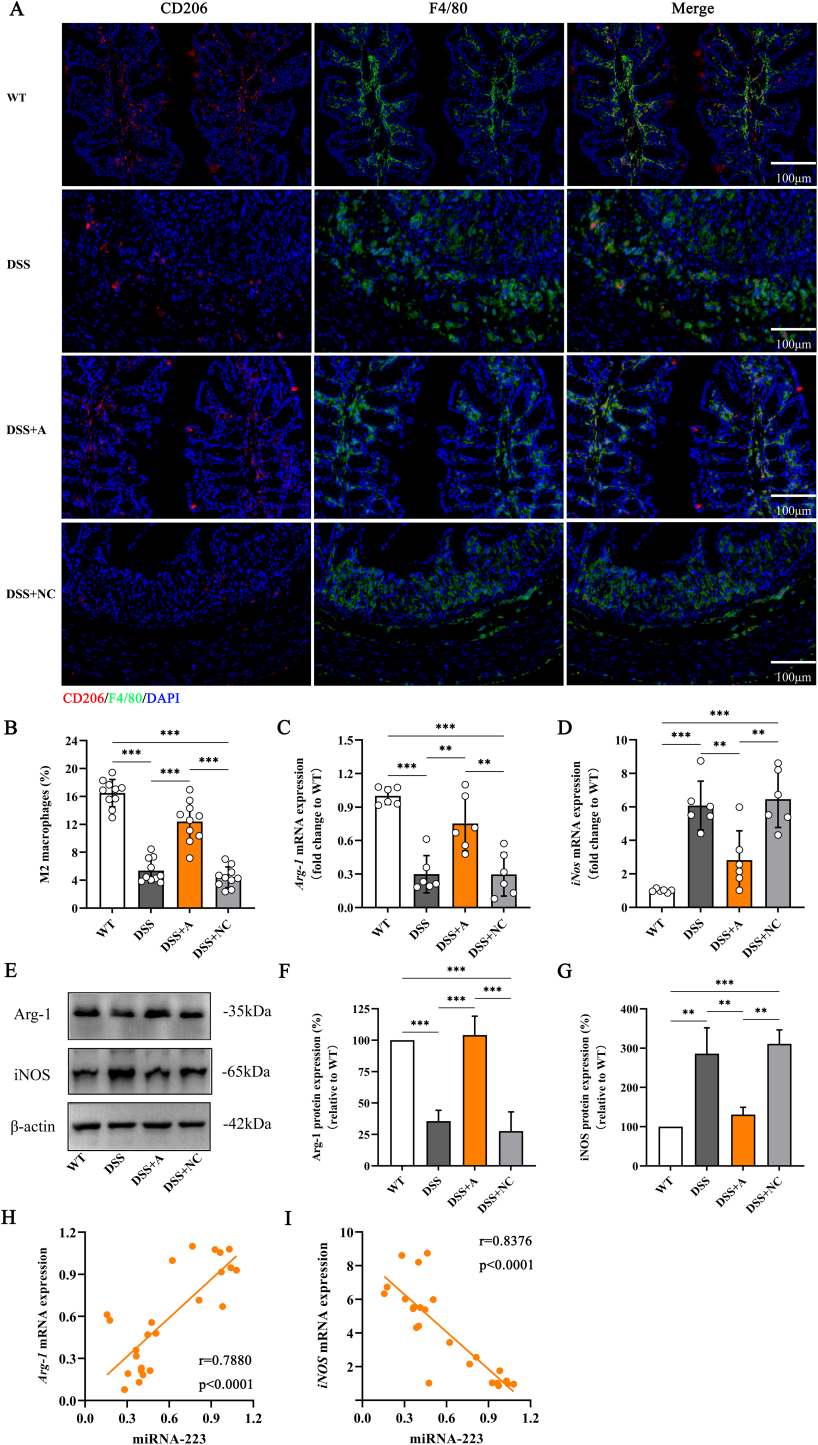
Increased polarization of M2 macrophages in DSS-induced colitis mice following administration of the miR-223 agomir. **(A)** Representative confocal laser scanning microscopy images showing the distributions of M2 macrophages (red) and total macrophages (blue) in colonic tissues. Scale bar: 100 µm. **(B)** Comparisons of the percentage of M2 macrophages among the groups. **(C)** Changes in the mRNA level of Arg-1, which is considered a specific biomarker of M2 macrophages, were analyzed *via* RT–qPCR. All the data are expressed as the means ± SDs (n=6 each group). *p<0.05, **p<0.01, ***p<0.001. **(D)** Changes in the mRNA level of iNOS, which is a specific biomarker of M2 macrophages. All the data are expressed as the means ± SDs (n=6 each group). *p<0.05, **p<0.01, ***p<0.001. **(E)** Representative western blot images showing the protein expression levels of Arg-1 and iNOS. The data represent the findings from three independent experiments. **(F, G)** Changes in the expression of Arg-1 and iNOS. **(H, I)** Associations between colonic miR-223 expression and Arg-1 or iNOS mRNA expression. Arg-1, arginase 1; iNOS, inducible nitric oxide synthase; RT–qPCR, quantitative reverse transcription PCR.

To investigate the role of miR-223 in macrophage polarization, we analyzed colonic macrophages using immunofluorescence for the pan-macrophage marker F4/80 and the M2 polarization marker CD206. Compared to the wild-type (WT) group, the DSS group exhibited a significant reduction in M2 macrophage polarization (**Figures 5A, B**). In contrast, miR-223 agomir treatment (DSS+A group) restored M2 polarization to levels surpassing those in the DSS group, indicating a shift toward anti-inflammatory macrophage phenotypes (**Figures 5A, B**).

We further quantified M1 and M2 polarization markers *via* RT-qPCR and western blotting. The DSS group showed elevated expression of inducible nitric oxide synthase (iNOS; an M1 marker) and reduced expression of arginase-1 (Arg-1; an M2 marker) compared to the WT group at both mRNA and protein levels (**Figures 5C–G**, **Supplementary Figures S1, S2**). Strikingly, miR-223 agomir administration reversed this imbalance: the DSS+A group displayed reduced iNOS and elevated Arg-1 expression compared to the DSS group (**Figures 5C–G**, **Supplementary Figures S1, S2**), confirming that miR-223 suppresses pro-inflammatory M1 polarization while promoting anti-inflammatory M2 polarization.

Correlation analyses reinforced these findings. miR-223 expression strongly positively correlated with Arg-1 mRNA levels (r = 0.7880, p < 0.0001; **Figure 5H**) and negatively correlated with iNOS mRNA levels (r = -0.8376, p < 0.0001; **Figure 5I**). These data underscore miR-223’s role in reprogramming macrophages toward an M2-dominant phenotype, thereby attenuating inflammation in colitis. Collectively, these results demonstrated that miR-223 could ameliorate DSS-induced colitis by skewing macrophage polarization from pro-inflammatory M1 to anti-inflammatory M2 states, highlighting its therapeutic potential in modulating immune responses.

### miR-223 drived macrophage M2 polarization *via* PPAR-γ/FOXO1 signaling

**Figure 6:**
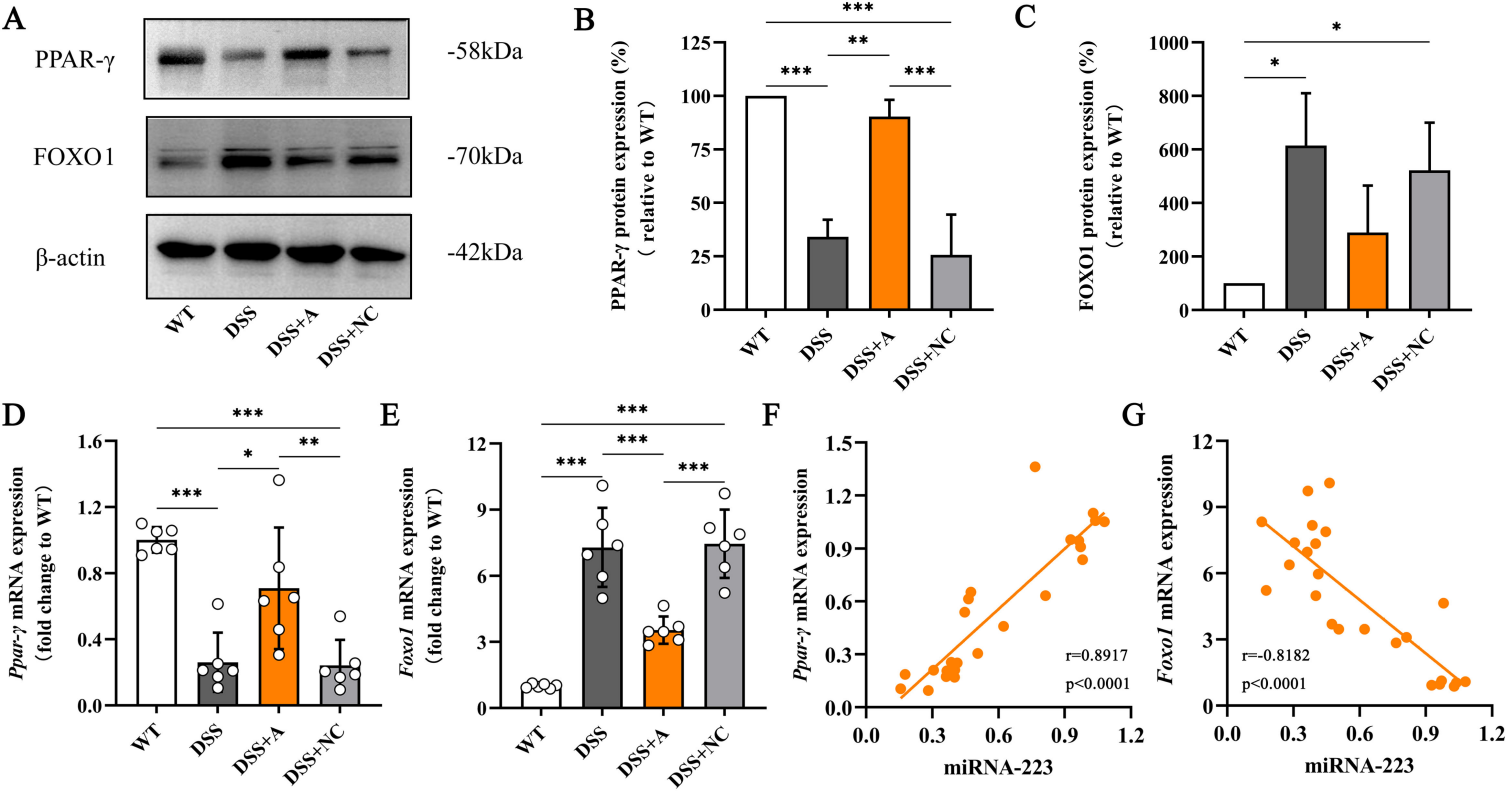
Relieving colitis by miR-223 through the promotion of macrophage M2 polarization *via* the modulation of PPAR-γ/FOXO1 signaling. **(A)** Representative images of western blots indicating the protein expression levels of PPAR-γ and FOXO1. The data represent the findings from three independent experiments. **(B, C)** Changes in the expression of PPAR-γ and FOXO1. **(D, E)** Changes in the mRNA levels of PPAR-γ and FOXO1. All the data are expressed as the means ± SDs (n=6 each group). *p<0.05, **p<0.01, ***p<0.001. **(F, G)** Correlations of colonic miR-223 expression with PPAR-γ and FOXO1 expression in the colon. Pearson analyses were used to correlate miR-223 with colonic PPAR-γ and FOXO1 expression. PPAR-γ, peroxisome proliferator-activated receptor gamma; FOXO1, Forkhead box transcription factor O1.

To delineate the molecular mechanism by which miR-223 promotes M2 polarization, we analyzed the PPAR-γ/FOXO1 signaling axis—a key regulator of macrophage phenotype switching. In the DSS group, colonic expression of FOXO1 (a transcriptional driver of pro-inflammatory M1 polarization) was significantly elevated, while PPAR-γ (a master regulator of M2 polarization) was reduced compared to the WT group (**Figures 6A–E**). miR-223 agomir treatment (DSS+A group) reversed these alterations, suppressing FOXO1 and restoring PPAR-γ expression to levels exceeding those in the DSS group (**Figures 6A–E**, **Supplementary Figures S3, S4**). Correlation analyses further substantiated miR-223’s regulatory role. miR-223 expression exhibited a strong positive correlation with PPAR-γ mRNA (r = 0.8917, p < 0.0001; **Figure 6F**) and a significant negative correlation with FOXO1 mRNA (r = -0.8182, p < 0.0001; **Figure 6G**).

### Expression of PPAR-γ and FOXO1 and the co-localization with macrophage

**Figure 7:**
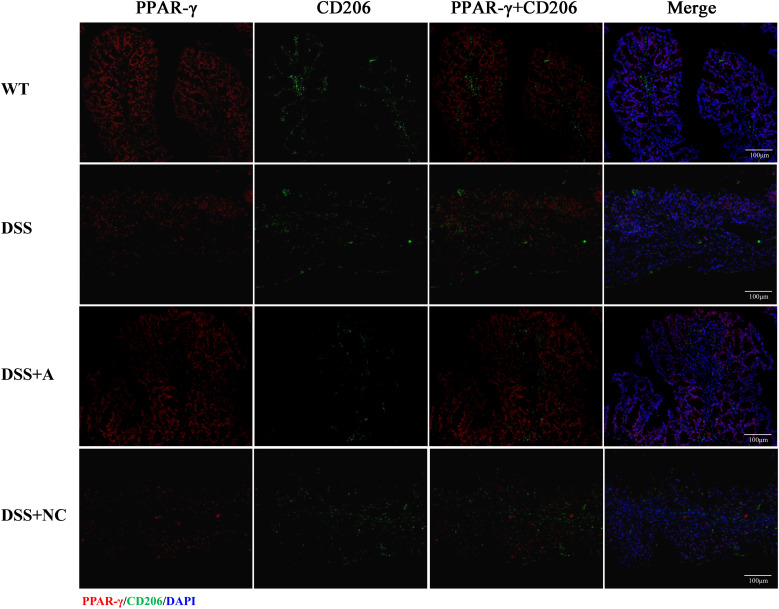
Immunofluorescence analysis of PPAR-γ and M2 macrophage marker CD206 in colonic tissue. Representative micrographs depict protein expression levels of PPAR-γ (red) and its co-localization (yellow) with CD206 (green) in colon sections. Nuclei were counterstained with DAPI (blue). Scale bar: 100 µm.

**Figure 8:**
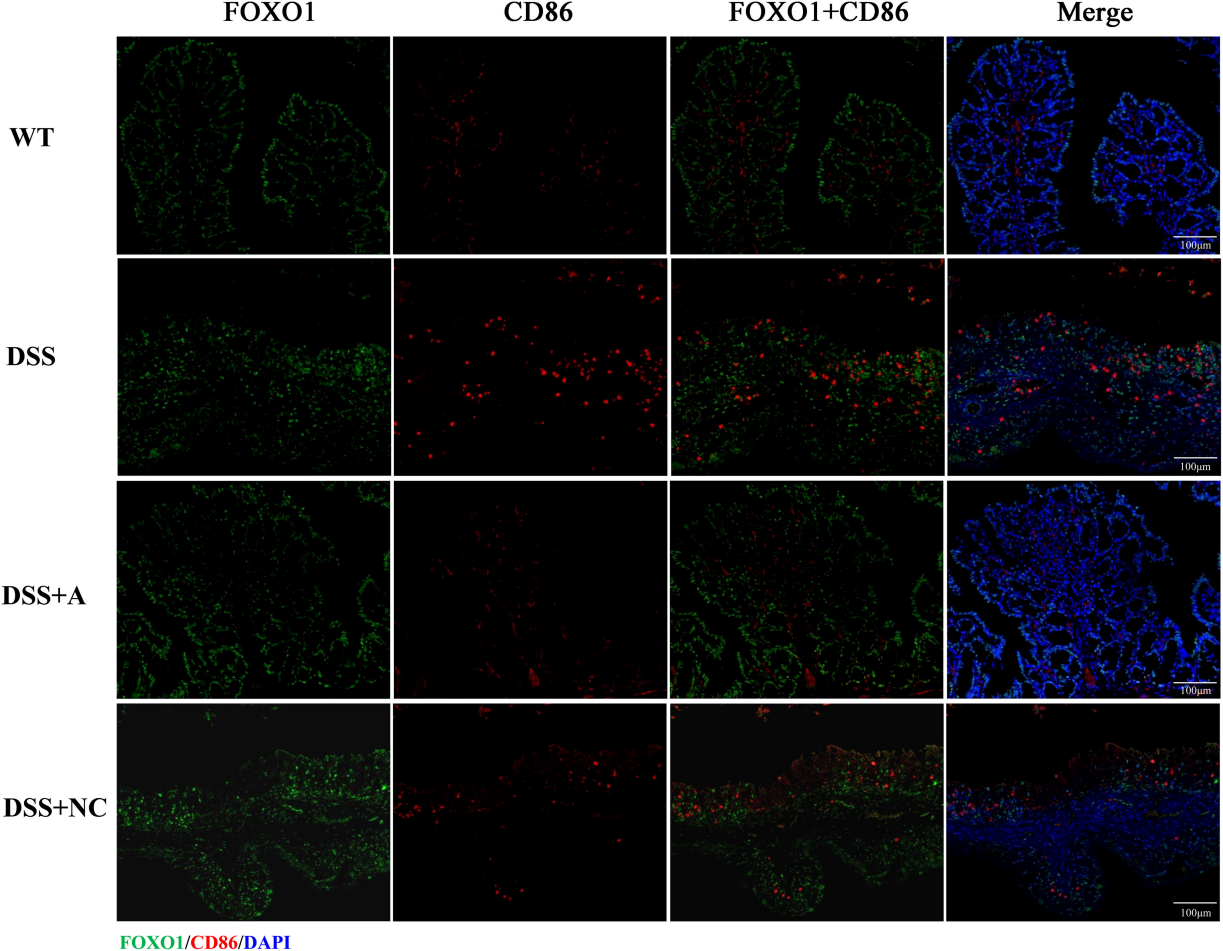
Immunofluorescence analysis of FOXO1 and M1 macrophage marker CD86 in colonic tissue. Representative micrographs depict protein expression levels of FOXO1 (green) and its co-localization (yellow) with CD86 (red) in colon sections. Nuclei were counterstained with DAPI (blue). Scale bar: 100 µm.

**Figure 9:**
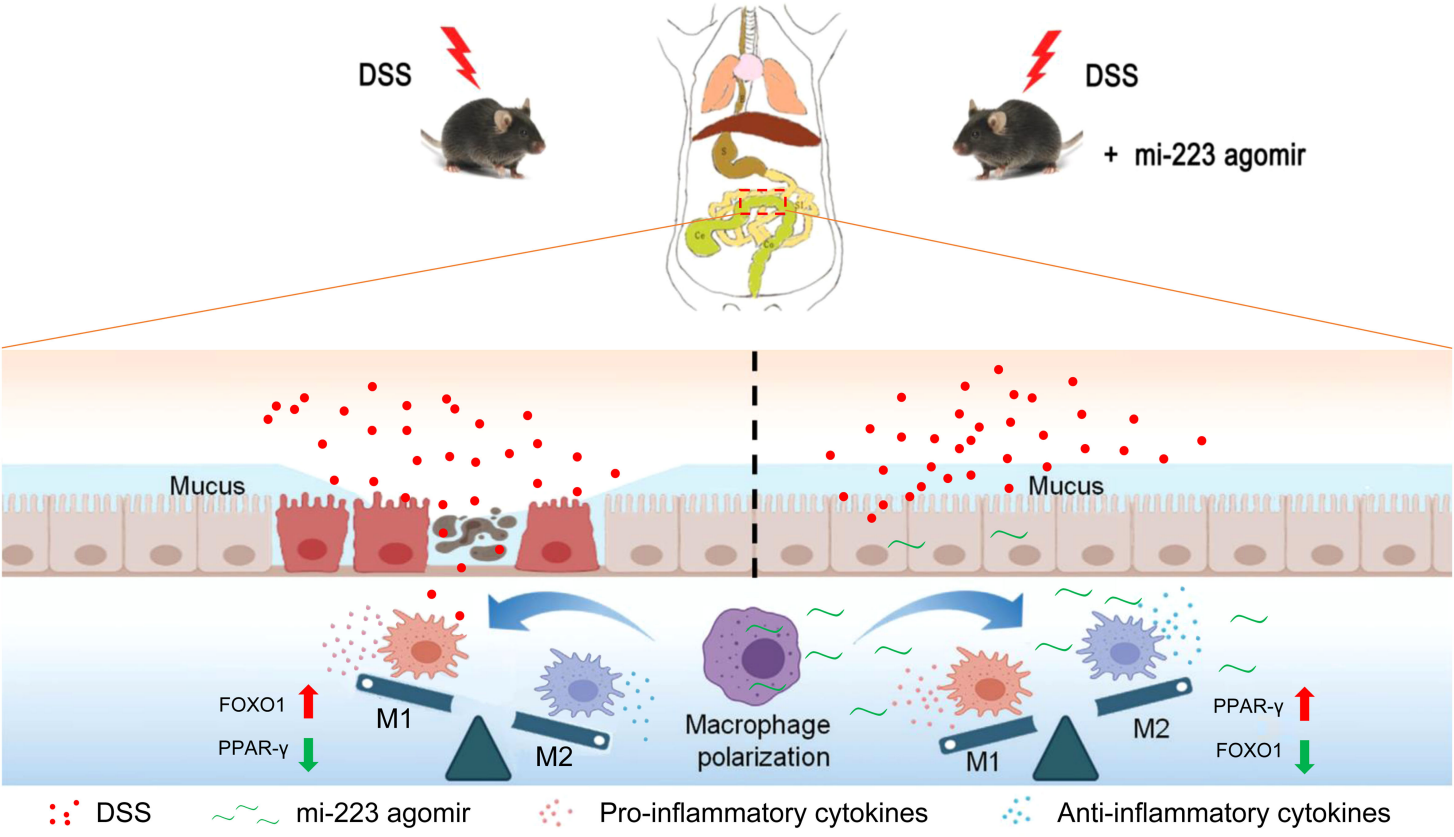
Schematic representation of the potential mechanisms underlying the targeted therapy of miR-223 supplement for DSS-induced colitis. miR-223 ameliorates DSS-induced colitis through promoting macrophage M2 polarization *via* modulation of PPAR-γ and FOXO1 signaling. DSS, dextran sodium sulfate.

We next investigated the expression and macrophage-specific localization of PPAR-γ and FOXO1. In DSS-induced colitis, colonic PPAR-γ expression decreased, while FOXO1 expression increased compared to WT controls (**Figures 7**, **8**). Treatment with miR-223 agomir (DSS+A group) reversed these alterations, restoring PPAR-γ expression toward WT levels and suppressing FOXO1 expression, consistent with our Western blot analyses. Importantly, PPAR-γ expression correlated positively with the M2 macrophage marker CD206, while FOXO1 expression correlated positively with the M1 macrophage marker CD86. Immunofluorescent co-staining confirmed this relationship, demonstrating specific co-localization of PPAR-γ with CD206 and FOXO1 with CD86 (**Figures 7**, **8**). Collectively, these findings suggest that miR-223 promoted PPAR-γ activity while suppressing FOXO1, thereby establishing a signaling balance favoring M2 macrophage polarization. By dual PPAR-γ/FOXO1 regulation, miR-223 shifted macrophages toward an anti-inflammatory M2 phenotype, attenuating colonic inflammation and tissue damage in DSS-induced colitis (**Figure 9**).

## Discussion

Macrophage polarization—a process that shapes immune responses by balancing pro-inflammatory (M1) and anti-inflammatory (M2) phenotypes—has emerged as a promising therapeutic target for inflammatory bowel disease (IBD). While miR-223 is known to influence macrophage behavior, its ability to alleviate colitis by modulating colonic macrophage polarization remained unexplored. In this study, using a DSS-induced murine colitis model and multi-modal analyses (RT-qPCR, western blotting, ELISA, immunofluorescence), we demonstrate that miR-223 agomir administration attenuates intestinal inflammation by reprogramming macrophage polarization through a novel dual mechanism. Specifically, miR-223 acts as a molecular switch, suppressing FOXO1 (a driver of M1 polarization) while upregulating PPAR-γ (a promoter of M2 polarization) (**Figure 9**). This shift reduces pro-inflammatory cytokines (TNF-α, IL-1β, IL-6), elevates anti-inflammatory IL-10, and mitigates mucosal damage, positioning miR-223 as a potent therapeutic candidate for ulcerative colitis (UC). Unlike prior studies in sepsis/metabolic disorders (10, 11), we demonstrate miR-223’s IBD-specific regulation of PPAR-γ/FOXO1 crosstalk. These findings not only elucidate a previously unrecognized regulatory axis (PPAR-γ/FOXO1) but also advance the translational potential of macrophage-targeted therapies in IBD, a field poised for clinical innovation.

IBD, characterized by chronic gastrointestinal inflammation, represents a growing global health burden with rising incidence worldwide. While its pathogenesis remains incompletely understood, IBD arises from a complex interplay of genetic susceptibility, environmental triggers, and dysregulated immune responses, particularly within the intestinal mucosa (16). Central to this dysregulation is the role of immune cells—including macrophages, T cells, and dendritic cells—that collectively mediate mucosal homeostasis or pathology (18). Among these, macrophages are critical sentinels positioned at the epithelial interface, where they orchestrate pathogen clearance, tissue repair, and immune tolerance (19). However, in IBD, an imbalance in macrophage polarization disrupts this equilibrium: pro-inflammatory M1 macrophages dominate, driving excessive cytokine production (*e.g.*, TNF-α, IL-6) and compromising the intestinal barrier, while anti-inflammatory M2 macrophages, which promote resolution and repair, are depleted (2, 3, 10, 20). This skewed M1/M2 ratio perpetuates inflammation and tissue damage, underscoring the therapeutic potential of restoring macrophage balance—either by suppressing M1 activation, enhancing M2 polarization, or both. Our findings align with this paradigm, demonstrating that miR-223 rectifies macrophage polarization imbalances, offering a targeted strategy to recalibrate intestinal immunity in IBD.

Macrophage polarization—a process influenced by microbial signals (*e.g*., lipopolysaccharide), cytokines, and miRNAs—serves as a critical checkpoint in inflammatory responses (2, 9). Among regulatory miRNAs, miR-223 has been implicated in promoting M2 polarization to resolve inflammation (10, 11), yet its role in IBD remained poorly defined. Here, we demonstrate that miR-223 agomir administration alleviates DSS-induced colitis by rebalancing macrophage phenotypes. In the DSS model, skewed polarization toward pro-inflammatory M1 macrophages disrupted intestinal immune homeostasis, amplifying pro-inflammatory cytokines (TNF-α, IL-1β, IL-6) and suppressing anti-inflammatory IL-10. miR-223 intervention reversed this imbalance, suppressing M1 polarization while enhancing M2 macrophages, thereby restoring cytokine equilibrium (**Figure 9**). These findings not only validate miR-223’s anti-inflammatory role in experimental colitis but also establish its mechanism: modulating macrophage polarization to resolve inflammation. By bridging this knowledge gap, our work expands the therapeutic potential of miRNA-driven macrophage reprogramming in IBD, offering a targeted strategy to correct immune dysregulation at its source.

Forkhead box transcription factor O1 (FOXO1), a transcription factor critical for mucosal immunity, regulates immune cell recruitment—including dendritic cells, macrophages, and neutrophils—and drives pro-inflammatory responses by promoting M1 macrophage polarization (21). In our DSS-induced colitis model, FOXO1 expression was significantly reduced compared to wild-type (WT) mice, a finding consistent with prior studies linking elevated FOXO1 activity to exacerbated intestinal inflammation (22). While this apparent contradiction (lower FOXO1 in a disease state) may reflect context-dependent roles of FOXO1 in inflammation, our data align with evidence that FOXO1 inhibition mitigates colitis severity. Crucially, we identified miR-223 as a direct suppressor of FOXO1, a relationship corroborated by earlier studies demonstrating miR-223’s targeting of FOXO1 in inflammatory contexts (23–25). By downregulating FOXO1, miR-223 curtails M1 macrophage polarization, thereby reducing pro-inflammatory cytokine production and attenuating colitis. This mechanism positions miR-223 as a key modulator of FOXO1-driven inflammation, offering a therapeutic avenue to disrupt pathological immune signaling in IBD.

Peroxisome proliferator-activated receptor gamma (PPAR-γ), a nuclear receptor highly expressed in the colon, serves as a critical regulator of intestinal inflammation and a promising therapeutic target for IBD (26, 27). Its ability to promote anti-inflammatory M2 macrophage polarization positions PPAR-γ as a key mediator of mucosal homeostasis (28, 29). Building on this, our study reveals that DSS-induced colitis suppresses PPAR-γ expression, while miR-223 agomir restores its levels, directly linking miR-223 to PPAR-γ-driven M2 polarization in IBD. This aligns with prior work by Ying et al., who identified miR-223 as a downstream effector of PPAR-γ in adipose tissue macrophages (30), suggesting a conserved regulatory axis across inflammatory contexts. Notably, the restoration of PPAR-γ expression by miR-223 may involve crosstalk with FOXO1 signaling. Wang et al. demonstrated that FOXO1 knockdown activates PPAR-γ in bone marrow-derived macrophages (31), mirroring our observation that miR-223 suppresses FOXO1 while enhancing PPAR-γ. This interplay suggests a novel mechanism: miR-223 may amplify PPAR-γ activity by targeting FOXO1, thereby synergistically promoting M2 polarization (**Figure 9**). While this hypothesis requires experimental validation, it highlights the unique capacity of miR-223 to concurrently inhibit pro-inflammatory (FOXO1/M1) and activate anti-inflammatory (PPAR-γ/M2) pathways, offering a dual-pronged strategy to rebalance macrophage dynamics in IBD.

The mature miR-223 sequence (UGUCAGUUUGUCAAAUACCCCA) is 100% conserved between mice (miRBase: MIMAT0000663) and humans (MIMAT0000280), highlighting its critical biological role and strong translational relevance. This sequence identity ensures consistent target gene regulation across species, as demonstrated by conserved functions in macrophage polarization and neutrophil activation. In a murine model with experimental autoimmune uveitis, miR-223 drives macrophages toward an anti-inflammatory (M2) phenotype by suppressing NLRP3/Notch signaling (32)—a mechanism confirmed in human IBD, where reduced miR-223 in inflamed tissues correlates with elevated IL-6 and TNF-α (33). Cross-species evidence from NAFLD/NASH further reveals that myeloid-derived miR-223 suppresses fibrotic genes (*e.g*., TAZ, NLRP3) in hepatocytes *via* exosomal transfer, with LDLR/APOE-dependent endocytosis facilitating uptake in both species (34, 35). These shared functions support therapeutic strategies for IBD, such as synthetic exosomes delivering miR-223 or repurposing PPARγ agonists (*e.g.*, pioglitazone) (36), which upregulate miR-223 and ameliorate inflammation in diabetic models and human cells (37). Critically, 100% human-mouse homology enables direct clinical translation of agomir-based therapies. Advances in nanoparticle engineering—such as encapsulating miR-223 agomirs—could further enhance macrophage-specific delivery, minimizing off-target effects and accelerating therapeutic development.

While our study offers novel insights into miR-223’s therapeutic potential in colitis, several limitations merit consideration. First, although we identified the FOXO1/PPAR-γ axis as a key mediator of miR-223’s effects on macrophage polarization, the upstream regulators and downstream effectors of this pathway require further mechanistic dissection. Ongoing studies employing FOXO1 inhibitors and PPAR-γ antagonists will help establish causality and refine our understanding of miR-223’s dual regulatory role. Second, while our findings in the DSS-induced colitis model are robust, complementary validation using human ulcerative colitis cell models (*e.g.*, patient-derived macrophages or organoids) and clinical cohorts is essential to strengthen translational relevance; such studies are currently underway. Third, although our whole-tissue analyses (WB, RT-qPCR) provided initial insights into the inflammatory milieu and miR-223’s effects, they reflect contributions from multiple cell types. While immunofluorescence data (**Figures 7**, **8**) specifically localizes PPAR-γ and FOXO1 modulation to macrophages, higher-resolution validation using flow-sorted macrophages or single-cell RNA sequencing would further confirm the macrophage-specificity of miR-223’s actions. Finally, the interplay between miR-223 and other key polarization regulators (*e.g.*, STAT6, NF-κB) remains unexplored. Systematic profiling of these interactions could reveal additional therapeutic targets. Future *in vitro* studies using macrophage cell lines should dissect miR-223’s dual regulation of PPAR-γ/FOXO1 crosstalk. Validation in chronic colitis models would further clarify miR-223’s long-term role in intestinal inflammation and macrophage dynamics. Addressing these gaps will solidify miR-223’s role in IBD and guide the development of macrophage-targeted, miRNA-based therapies.

In conclusion, this study provides the first experimental demonstration that miR-223 supplementation exerts potent anti-inflammatory effects in DSS-induced colitis, significantly attenuating mucosal damage and inflammation. The therapeutic efficacy could stem from its dual regulatory capacity to shift macrophage polarization: suppressing M1 pro-inflammatory phenotype *via* FOXO1 downregulation, and activating M2 anti-inflammatory responses through PPAR-γ upregulation, thereby establishing the FOXO1/PPAR-γ axis as the mechanistic cornerstone of miR-223’s action. Our findings position miR-223 as a promising therapeutic agent for UC through its unique ability to simultaneously target macrophage plasticity and epithelial microenvironment. The identified PPAR-γ/FOXO1 signaling nexus not only clarifies colitis pathogenesis but also provides a druggable pathway for precision immunomodulation. Our discovery of this miR-223-mediated crosstalk between transcriptional regulators fundamentally advances understanding of IBD pathogenesis and establishes macrophage reprogramming as a viable epigenetic strategy for inflammatory diseases. While focused on acute colitis models, these findings implicate the potential role of miR-223 in chronic intestinal inflammation, warranting further investigation in spontaneous colitis models and clinical-grade delivery systems to accelerate translational applications.

## Data Availability

The original contributions presented in the study are included in the article/**Supplementary Material**, further inquiries can be directed to the corresponding author/s.

## References

[1] Li F Huang H Zhao P Jiang J Ding X Lu D. Curculigoside mitigates dextran sulfate sodium-induced colitis by activation of KEAP1-NRF2 interaction to inhibit oxidative damage and autophagy of intestinal epithelium barrier. Int J Mol Med. (2023) 52:107. doi: 10.3892/ijmm.2023.5310, PMID: 37772380 PMC10558217

[2] Zhang K Guo J Yan W Xu L. Macrophage polarization in inflammatory bowel disease. Cell Commun Signal. (2023) 21:367. doi: 10.1186/s12964-023-01386-9, PMID: 38129886 PMC10734116

[3] Du Y Rong L Cong Y Shen L Zhang N Wang B. Macrophage polarization: an effective approach to targeted therapy of inflammatory bowel disease. Expert Opin Ther Targets. (2021) 25:191–209. doi: 10.1080/14728222.2021.1901079, PMID: 33682588

[4] Sun Y Li H Duan X Ma X Liu C Shang D. Chensinin-1b alleviates DSS-induced inflammatory bowel disease by inducing macrophage switching from the M1 to the M2 phenotype. Biomedicines. (2024) 12:345. doi: 10.3390/biomedicines12020345, PMID: 38397947 PMC10886634

[5] Yang Y Zhao C Yang Z Du C Chang Z Wen X. Myeloid-derived growth factor ameliorates dextran sodium sulfate-induced colitis by regulating macrophage polarization. J Mol Med (Berl). (2024) 102:875–86. doi: 10.1007/s00109-024-02447-3, PMID: 38695882 PMC11213757

[6] Zhu W Chen Q Li Y Wan J Li J Tang S. HIF-1α-overexpressing mesenchymal stem cells attenuate colitis by regulating M1-like macrophages polarization toward M2-like macrophages. Biomedicines. (2023) 11:825. doi: 10.3390/biomedicines11030825, PMID: 36979804 PMC10045413

[7] Qiu W Akanyibah FA Xia Y Ocansey DKW Mao F Liang Y. Emerging role of small RNAs in inflammatory bowel disease and associated colorectal cancer (Review). Int J Mol Med. (2025) 55:33. doi: 10.3892/ijmm.2024.5474, PMID: 39704210 PMC11670865

[8] Jiao P Wang XP Luoreng ZM Yang J Jia L Ma Y. miR-223: an effective regulator of immune cell differentiation and inflammation. Int J Biol Sci. (2021) 17:2308–22. doi: 10.7150/ijbs.59876, PMID: 34239357 PMC8241730

[9] Lu H Suo Z Lin J Cong Y Liu Z. Monocyte-macrophages modulate intestinal homeostasis in inflammatory bowel disease. biomark Res. (2024) 12:76. doi: 10.1186/s40364-024-00612-x, PMID: 39095853 PMC11295551

[10] Yuan S Wu Q Wang Z Che Y Zheng S Chen Y. miR-223: an immune regulator in infectious disorders. Front Immunol. (2021) 12:781815. doi: 10.3389/fimmu.2021.781815, PMID: 34956210 PMC8702553

[11] Chen X Wu Y Li R Li C Xu L Qiao W. Galactose-modified nanoparticles for delivery of microRNA to mitigate the progress of abdominal aortic aneurysms by regulating macrophage polarization. Nanomedicine. (2022) 44:102564. doi: 10.1016/j.nano.2022.102564, PMID: 35643269

[12] Zhang J Wang C Guo Z Da B Zhu W Li Q. miR-223 improves intestinal inflammation through inhibiting the IL-6/STAT3 signaling pathway in dextran sodium sulfate-induced experimental colitis. Immun Inflammation Dis. (2021) 9:319–27. doi: 10.1002/iid3.395, PMID: 33332758 PMC7860526

[13] Zhou H Xiao J Wu N Liu C Xu J Liu F. MicroRNA-223 regulates the differentiation and function of intestinal dendritic cells and macrophages by targeting C/EBPβ. Cell Rep. (2015) 13:1149–60. doi: 10.1016/j.celrep.2015.09.073, PMID: 26526992

[14] Neudecker V Haneklaus M Jensen O Khailova L Masterson JC Tye H. Myeloid-derived miR-223 regulates intestinal inflammation *via* repression of the NLRP3 inflammasome. J Exp Med. (2017) 214:1737–52. doi: 10.1084/jem.20160462, PMID: 28487310 PMC5460990

[15] Zhang N Fu L Bu Y Yao Y Wang Y. Downregulated expression of miR-223 promotes Toll-like receptor-activated inflammatory responses in macrophages by targeting RhoB. Mol Immunol. (2017) 91:42–8. doi: 10.1016/j.molimm.2017.08.026, PMID: 28881218

[16] Yu X Li X Xu Y Li Y Zhou Y Zhang J. Resveratrol ameliorates ulcerative colitis by upregulating Nrf2/HO-1 pathway activity: integrating animal experiments and network pharmacology. Mol Med Rep. (2024) 29:77. doi: 10.3892/mmr.2024.13201, PMID: 38488031

[17] Neurath MF Fuss I Kelsall BL Stüber E Strober W. Antibodies to interleukin 12 abrogate established experimental colitis in mice. J Exp Med. (1995) 182:1281–90. doi: 10.1084/jem.182.5.1281, PMID: 7595199 PMC2192205

[18] Mowat AM Agace WW. Regional specialization within the intestinal immune system. Nat Rev Immunol. (2014) 14:667–85. doi: 10.1038/nri3738, PMID: 25234148

[19] Chang JT. Pathophysiology of inflammatory bowel diseases. N Engl J Med. (2020) 383:2652–64. doi: 10.1056/NEJMra2002697, PMID: 33382932

[20] Yunna C Mengru H Lei W Weidong C. Macrophage M1/M2 polarization. Eur J Pharmacol. (2020) 877:173090. doi: 10.1016/j.ejphar.2020.173090, PMID: 32234529

[21] Graves DT Milovanova TN. Mucosal immunity and the FOXO1 transcription factors. Front Immunol. (2019) 10:2530. doi: 10.3389/fimmu.2019.02530, PMID: 31849924 PMC6896163

[22] Han C Guo L Sheng Y Yang Y Wang J Gu Y. FoxO1 regulates TLR4/MyD88/MD2-NF-κB inflammatory signaling in mucosal barrier injury of inflammatory bowel disease. J Cell Mol Med. (2020) 24:3712–23. doi: 10.1111/jcmm.15075, PMID: 32057181 PMC7131908

[23] Li Y Deng S Peng J Wang X Essandoh K Mu X. MicroRNA-223 is essential for maintaining functional β-cell mass during diabetes through inhibiting both FOXO1 and SOX6 pathways. J Biol Chem. (2019) 294:10438–48. doi: 10.1074/jbc.RA119.007755, PMID: 31118273 PMC6615686

[24] Wu L Li H Jia CY Cheng W Yu M Peng M. MicroRNA-223 regulates FOXO1 expression and cell proliferation. FEBS Lett. (2012) 586:1038–43. doi: 10.1016/j.febslet.2012.02.050, PMID: 22569260

[25] Han LL Zhou XJ Li FJ Hao XW Jiang Z Dong Q. MiR-223-3p promotes the growth and invasion of neuroblastoma cell by targeting FOXO1. Eur Rev Med Pharmacol Sci. (2019) 23:8984–90. doi: 10.26355/eurrev_201910_19298, PMID: 31696486

[26] Venkataraman B Ojha S Belur PD Bhongade B Raj V Collin PD. Phytochemical drug candidates for the modulation of peroxisome proliferator-activated receptor γ in inflammatory bowel diseases. Phytother Res. (2020) 34:1530–49. doi: 10.1002/ptr.6625, PMID: 32009281

[27] Decara J Rivera P López-Gambero AJ Serrano A Pavón FJ Baixeras E. Peroxisome proliferator-activated receptors: experimental targeting for the treatment of inflammatory bowel diseases. Front Pharmacol. (2020) 11:730. doi: 10.3389/fphar.2020.00730, PMID: 32536865 PMC7266982

[28] Toobian D Ghosh P Katkar GD. Parsing the role of PPARs in macrophage processes. Front Immunol. (2021) 12:783780. doi: 10.3389/fimmu.2021.783780, PMID: 35003101 PMC8727354

[29] Yu L Gao Y Aaron N Qiang L. A glimpse of the connection between PPAR-γ and macrophage. Front Pharmacol. (2023) 14:1254317. doi: 10.3389/fphar.2023.1254317, PMID: 37701041 PMC10493289

[30] Ying W Tseng A Chang RC Morin A Brehm T Triff K. MicroRNA-223 is a crucial mediator of PPAR-γ-regulated alternative macrophage activation. J Clin Invest. (2015) 125:4149–59. doi: 10.1172/JCI81656, PMID: 26436647 PMC4639972

[31] Wang Z Luo W Zhao C Yu M Li H Zhou F. FoxO1-modulated macrophage polarization regulates osteogenesis via PPAR-γ signaling. Biochim Biophys Acta Mol Basis Dis. (2024) 187:167333. doi: 10.1016/j.bbadis.2024.167333, PMID: 38960054

[32] Qu R Peng Y Zhou M Xu S Yin X Qiu Y. MiR-223-3p attenuates M1 macrophage polarization *via* suppressing the Notch signaling pathway and NLRP3-mediated pyroptosis in experimental autoimmune uveitis. Eur J Pharmacol. (2023) 960:176139. doi: 10.1016/j.ejphar.2023.176139, PMID: 38059448

[33] Liu L Dong Y Ye M Jin S Yang J Joosse ME. The pathogenic role of nlrp3 inflammasome activation in inflammatory bowel diseases of both mice and humans. J Crohns Colitis. (2017) 11:737–50. doi: 10.1093/ecco-jcc/jjw219, PMID: 27993998 PMC5881697

[34] Hou X Yin S Ren R Liu S Yong L Liu Y. Myeloid-cell-specific il-6 signaling promotes microrna-223-enriched exosome production to attenuate nafld-associated fibrosis. Hepatology. (2021) 74:116–32. doi: 10.1002/hep.31658, PMID: 33236445 PMC8141545

[35] He Y Rodrigues RM Wang X Seo W Ma J Hwang S. Neutrophil-to-hepatocyte communication *via* LDLR-dependent miR-223-enriched extracellular vesicle transfer ameliorates nonalcoholic steatohepatitis. J Clin Invest. (2021) 131:e141513. doi: 10.1172/JCI141513, PMID: 33301423 PMC7843220

[36] Iftikhar QUA Iftikhar MK Iqbal J Sathian B. Balancing promise and uncertainty: PPAR agonists in ibd therapy. J Gastroenterol Hepatol. (2015) 40:1646–7. doi: 10.1111/jgh.16978, PMID: 40259682

[37] Joharapurkar A Patel V Kshirsagar S Patel MS Savsani H Jain M. Effect of dual PPAR-α/γ agonist saroglitazar on diabetic retinopathy and oxygen-induced retinopathy. Eur J Pharmacol. (2021) 899:174032. doi: 10.1016/j.ejphar.2021.174032, PMID: 33753107

