# Supplementary material for: miR-223 alleviates DSS-induced colitis by prompting macrophage M2 polarization through PPAR-γ/FOXO1 signaling
Source: Front Immunol. 2025 Jul 29;16:1598781. doi: 10.3389/fimmu.2025.1598781 (PMC12339350; doi:10.3389/fimmu.2025.1598781)

**Supplementary Materials**

**Figure S1.** Original Western blot images for Arg-1 (Arg-1, ~35 kDa). Left: Merged image with targeted Arg-1 band indicated by the arrow. Right: Background control image. Lane designations: M: Protein molecular weight markers; Lane 1: Wild-type (WT) control; Lane 2: DSS-treated group (DSS); Lane 3: DSS+miR-223 agomir (DSS+A); Lane 4: DSS+miR-223 agomir negative control (DSS+NC).

**Replicate 1:**

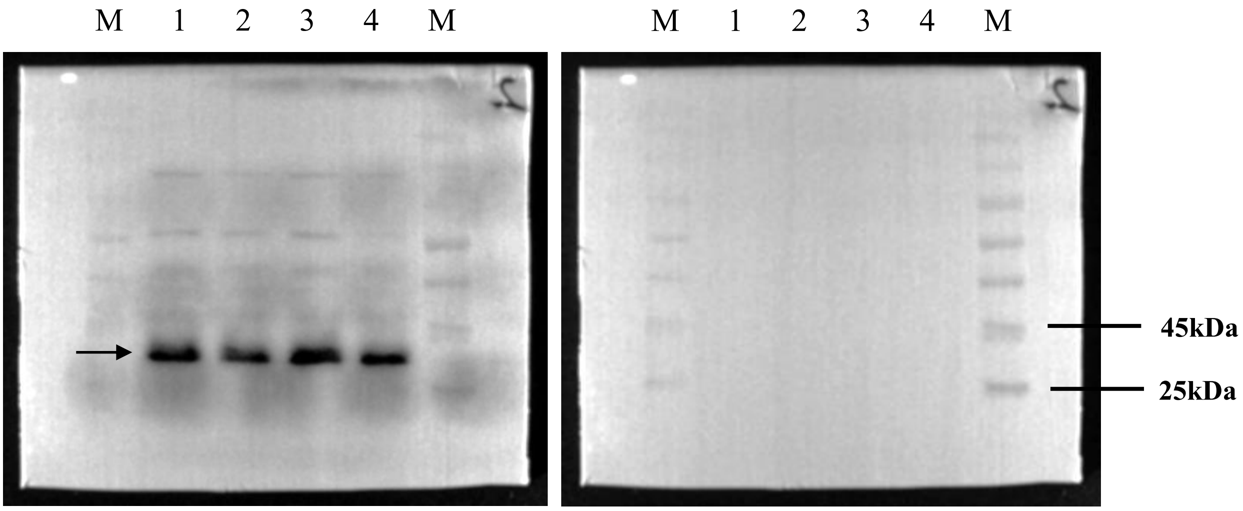


**Replicate 2:**

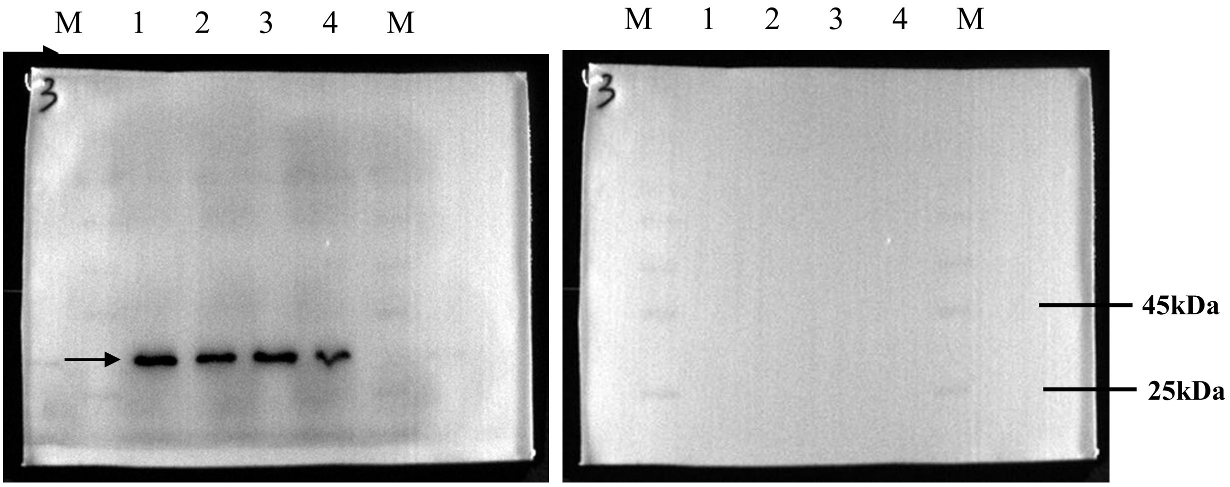

**Replicate 3:**

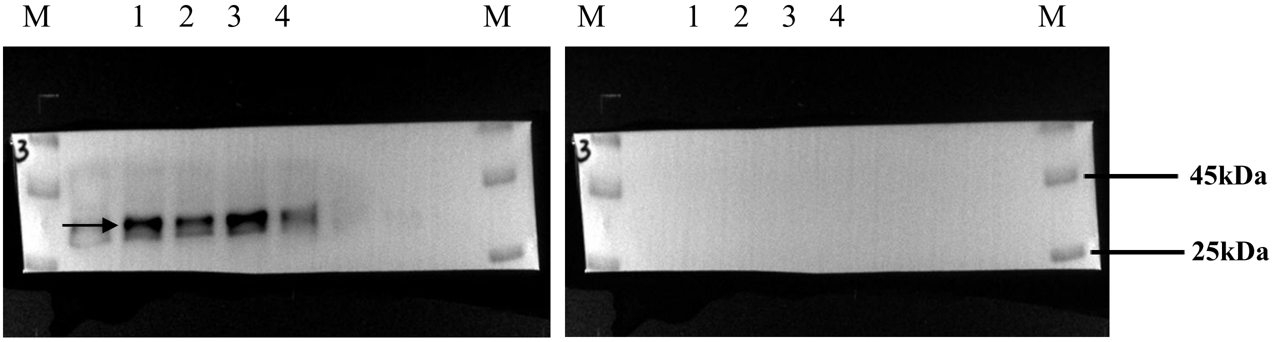


**Figure S2.** Original Western blot images for iNOS (～65kDa). Left: Merged image with targeted iNOS band indicated by the arrow. Right: Background control image. Lane designations: M: Protein molecular weight markers; Lane 1: Wild-type (WT) control; Lane 2: DSS-treated group (DSS); Lane 3: DSS+miR-223 agomir (DSS+A); Lane 4: DSS+miR-223 agomir negative control (DSS+NC).

**Replicate 1：**

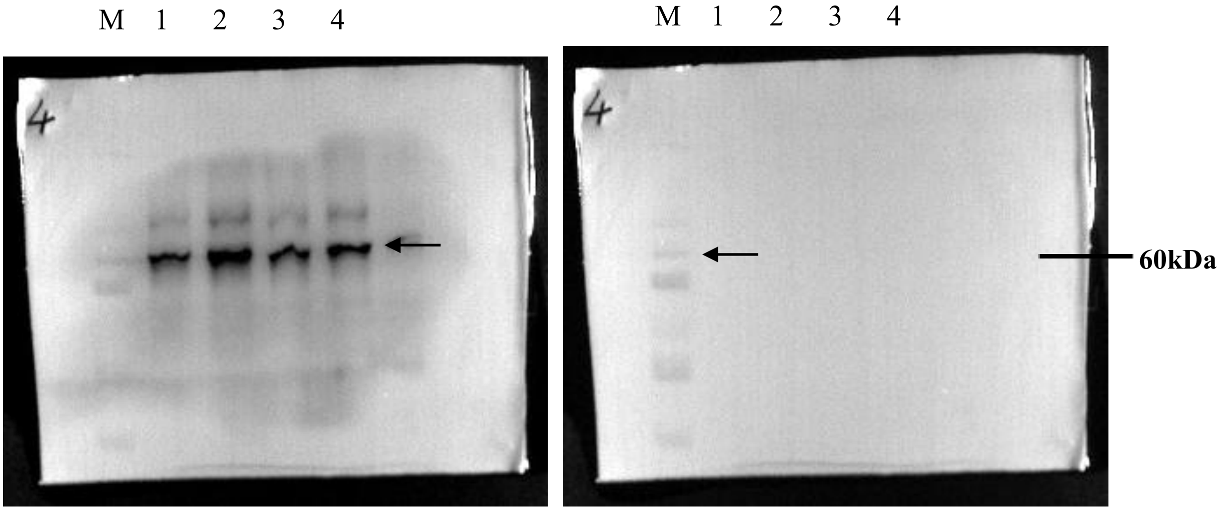

**Replicate 2:**

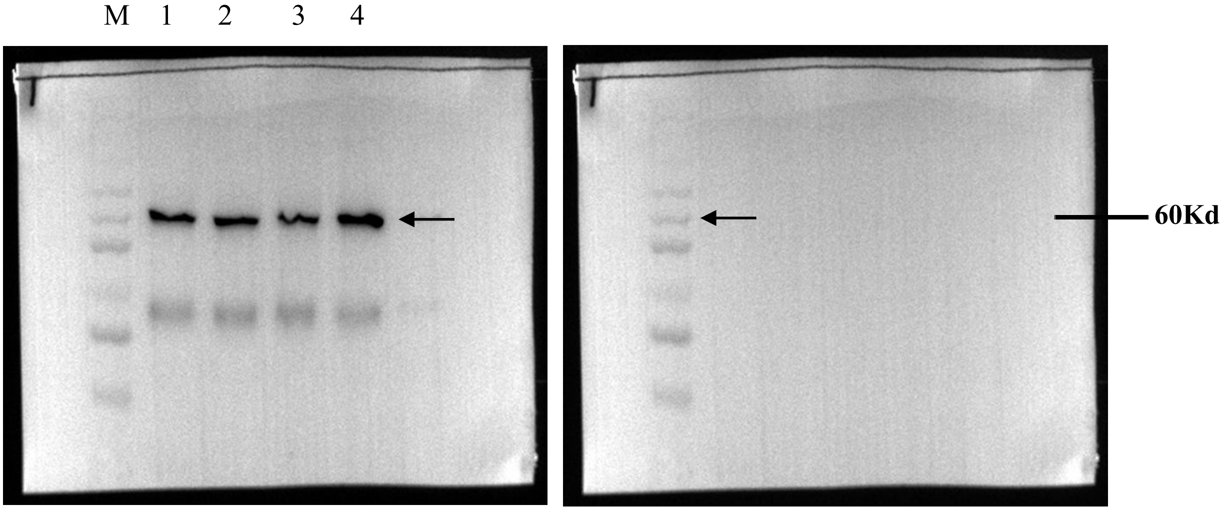


**Replicate 3:**

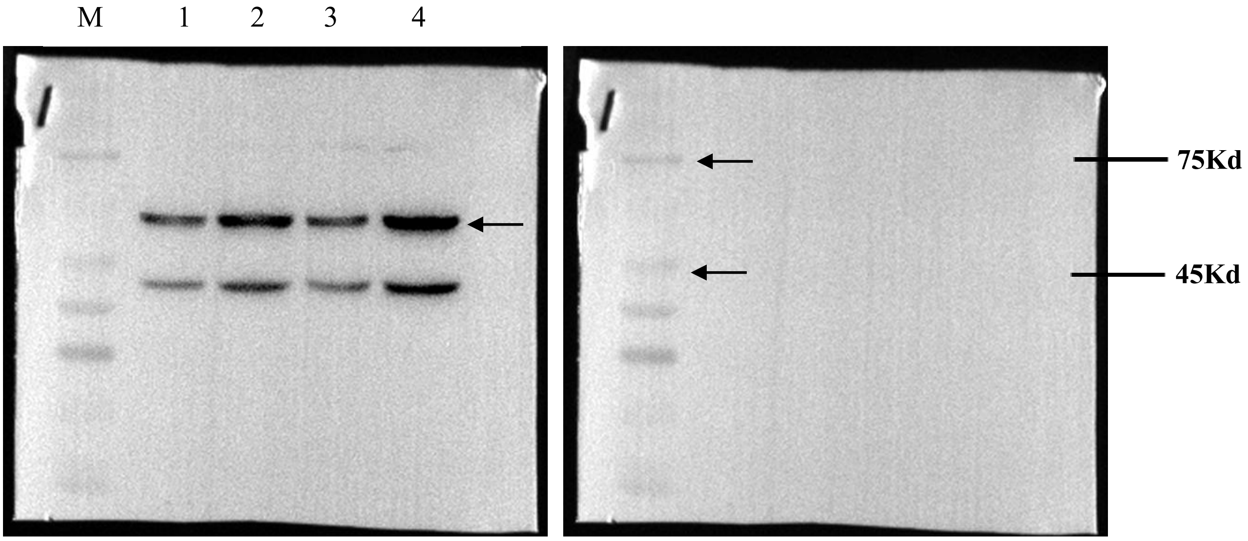

**Figure S3.** Original Western blot images for PPAR-γ (～58kDa). Left: Merged image with targeted PPAR-γ band indicated by the arrow. Right: Background control image. Lane designations: M: Protein molecular weight markers; Lane 1: Wild-type (WT) control; Lane 2: DSS-treated group (DSS); Lane 3: DSS+miR-223 agomir (DSS+A); Lane 4: DSS+miR-223 agomir negative control (DSS+NC).

**Replicate 1:**

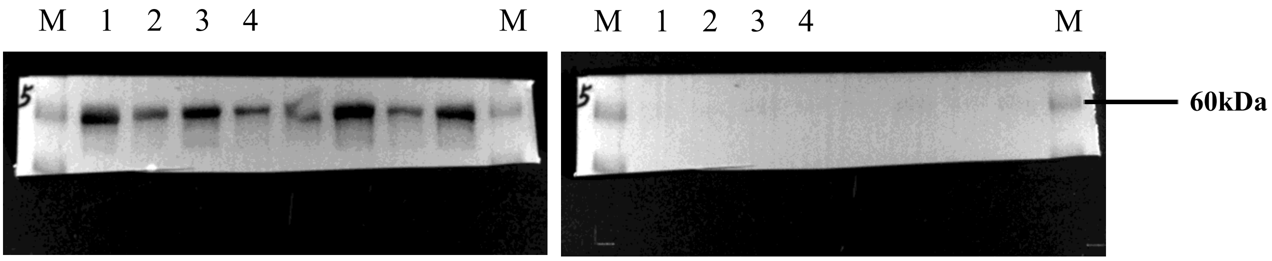


**Replicate 2:**

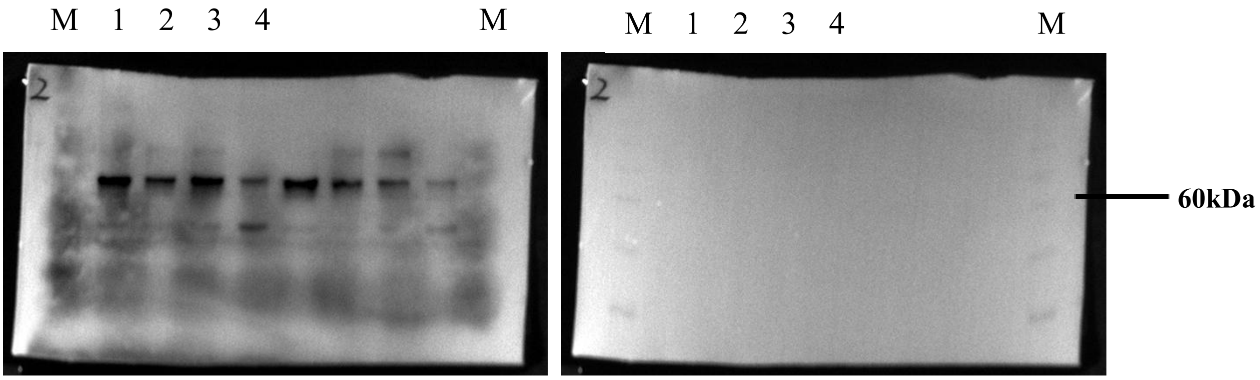


**Replicate 3:**

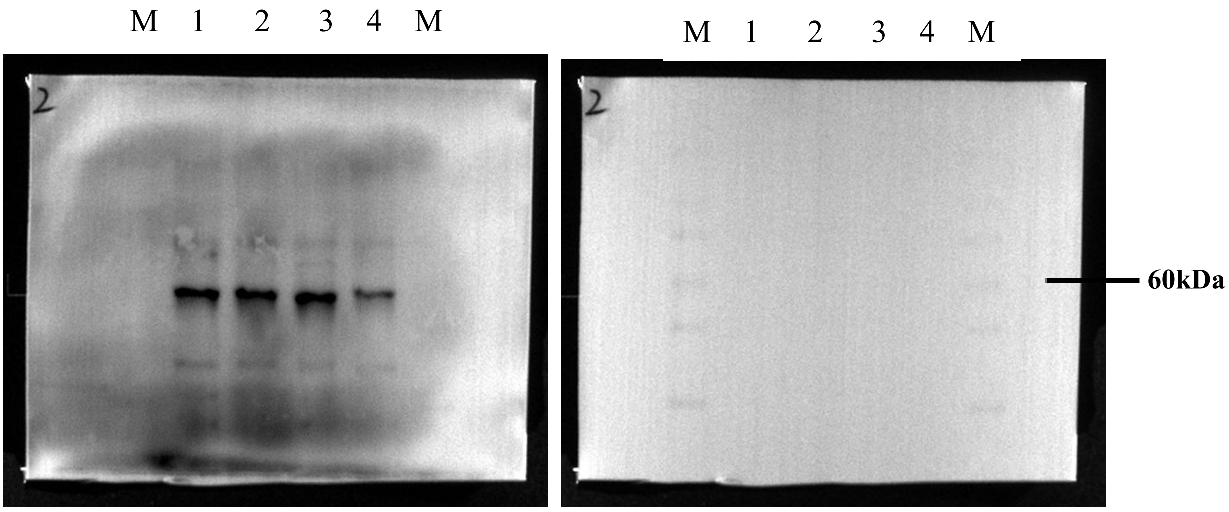

**Figure S4.** Original Western blot images for FOXO1 (～70kDa). Left: Merged image with targeted FOXO1 band indicated by the arrow. Right: Background control image. Lane designations: M: Protein molecular weight markers; Lane 1: Wild-type (WT) control; Lane 2: DSS-treated group (DSS); Lane 3: DSS+miR-223 agomir (DSS+A); Lane 4: DSS+miR-223 agomir negative control (DSS+NC).

**Replicate 1:**

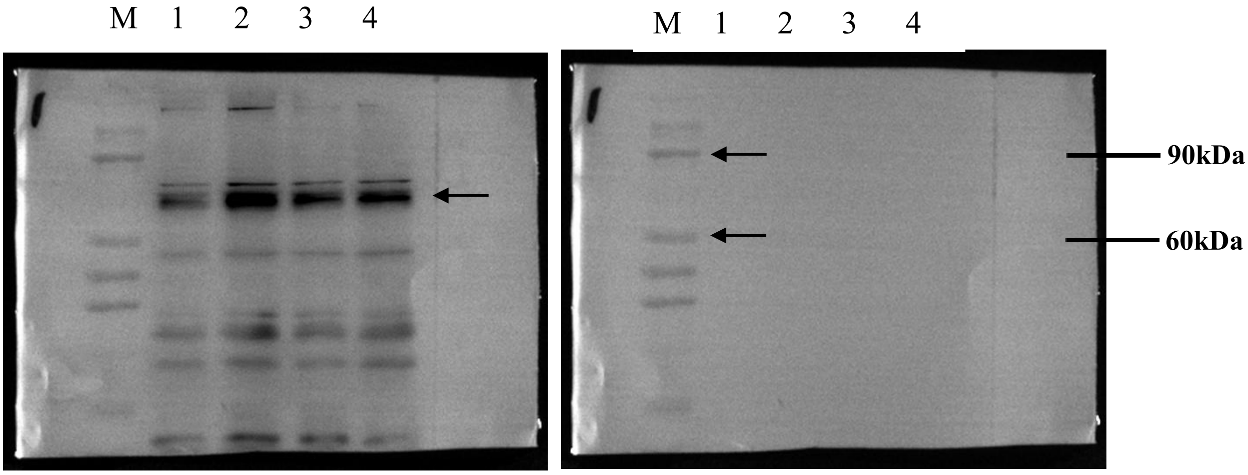


**Replicate 2:**

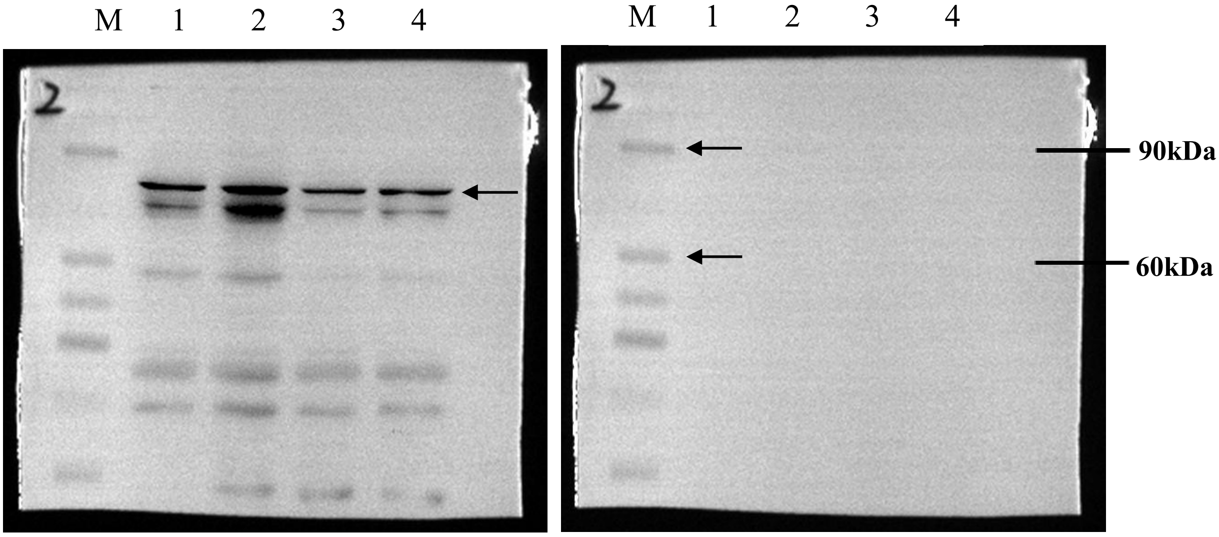

**Replicate 3:**

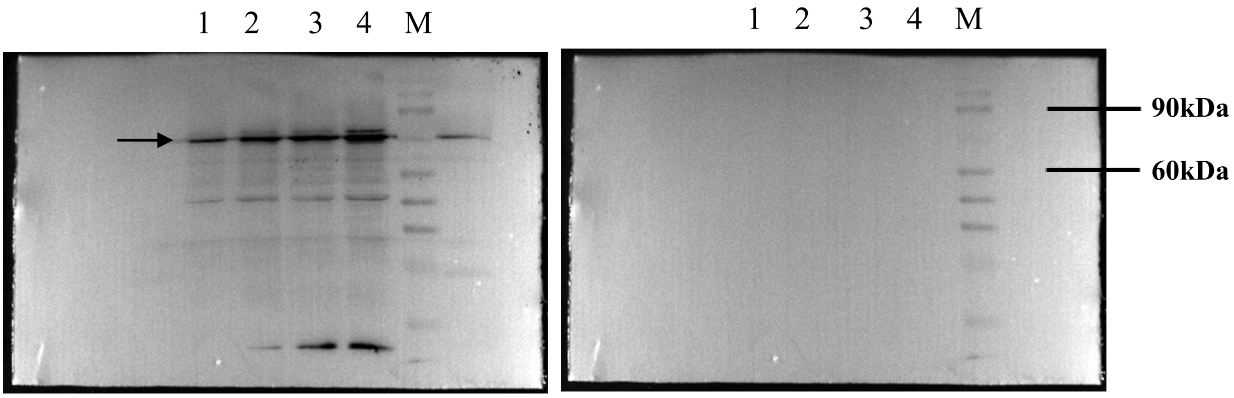


**Figure S5.** Original Western blot images for β-actin (~42kDa). Left: Merged image with targeted β-actin band indicated by the arrow. Right: Background control image. Lane designations: M: Protein molecular weight markers; Lane 1: Wild-type (WT) control; Lane 2: DSS-treated group (DSS); Lane 3: DSS+miR-223 agomir (DSS+A); Lane 4: DSS+miR-223 agomir negative control (DSS+NC).

**Replicate 1:**

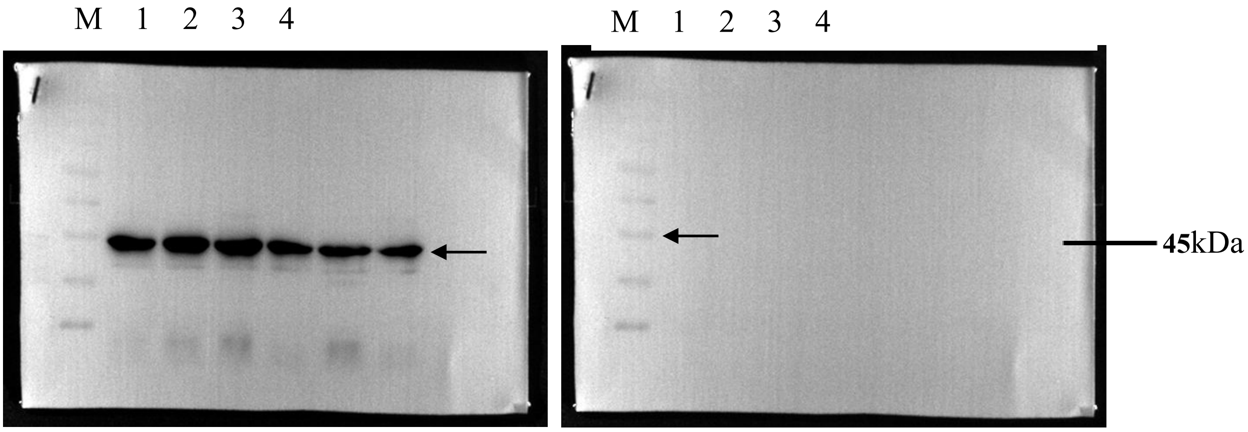


**Replicate 2:**

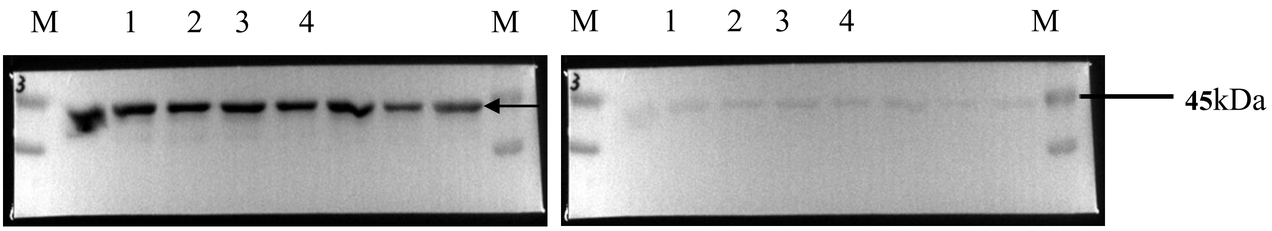

**Replicate 3:**

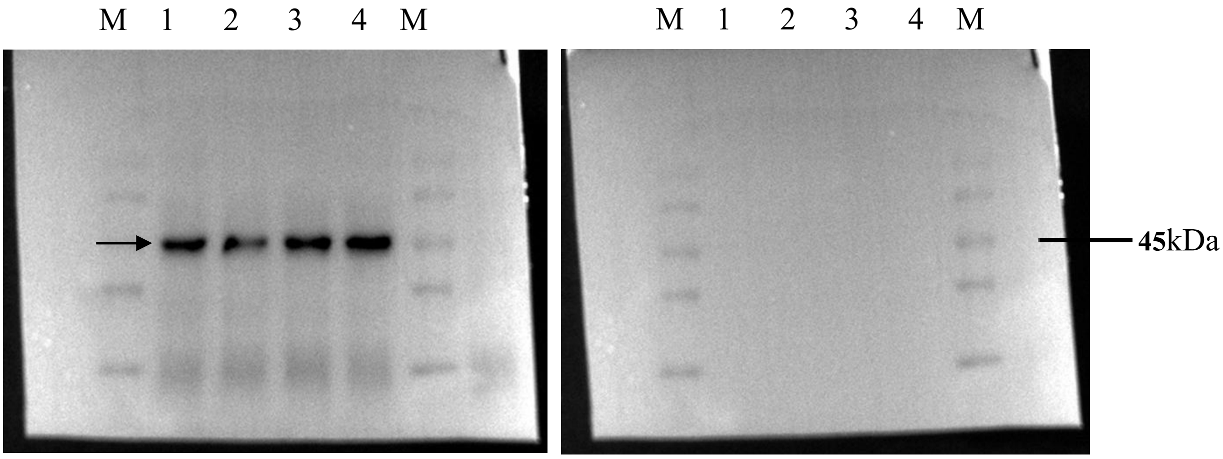

Supplement: Supplementary file 1 [file DataSheet1.pdf]
